# Adolescent mental health help-seeking behaviours in rural Australia: cross-sectional analysis of a nationwide cohort study

**DOI:** 10.1186/s13034-026-01022-7

**Published:** 2026-02-04

**Authors:** Ali Ahmed, Riaz Uddin, Allen G. Ross, Shannon Edmed, Anayochukwu E. Anyasodor, Subash Thapa, Kedir Y. Ahmed, Catherine Keniry, Feleke H. Astawesegn, Mahmood Shakeel, Simon S. Smith, M. Mamun Huda

**Affiliations:** 1 Rural Health Research Institute, Charles Sturt University, Orange, NSW Australia; 2 Child Health Research Centre, The University of Queensland, Brisbane, QLD Australia; 3 The Australian Research Council Centre of Excellence for Children and Families over the Life Course, The University of Queensland, Brisbane, QLD Australia; 4 School of Rural Medicine, Charles Sturt University, Orange, NSW Australia

**Keywords:** Adolescent mental health, Psychological health, Help-seeking behaviour, Rural health services, Australia

## Abstract

**Background:**

Adolescent mental health outcomes are often poorer in rural areas of Australia, and most adolescents do not seek help, highlighting a critical gap in understanding help-seeking behaviours. This study examined mental health help-seeking patterns and associated factors among rural Australian adolescents.

**Methods:**

Data from Wave 8 of the Longitudinal Study of Australian Children, including 4,837 adolescents aged 14–19 years, were analysed. The prevalence of help-seeking overall and by remoteness, as defined by the Australian Bureau of Statistics were estimated. Cluster-adjusted multiple logistic regression models were used to examine factors associated with help-seeking behaviours.

**Results:**

Help-seeking behaviours were generally lower among adolescents from rural areas compared to their urban counterparts. Seeking face-to-face mental health professional help was significantly less common in outer regional and remote areas (7.72%, 95% CI: 5.39–10.93) compared to urban areas (12.20%, 10.97–13.54). Furthermore, males reported significantly lower professional help-seeking behaviours (2.76%, 1.33–5.63) than females (13.53%, 9.08–19.70) in outer regional and remote areas. Similar sex disparities were observed in non-face-to-face (e.g., internet, phone) help-seeking. The most common predictors of help-seeking behaviours were ongoing anxiety or depression and good parent-child relationships. Other statistically significant predictors included suicidal thoughts and behaviours, single-parent family, community participation, social media exposure and drug use. Two predictors (i.e., financial hardship for formal help-seeking and community engagement for informal help-seeking) varied statistically significantly between rural and urban settings.

**Conclusion:**

Strategies to address lower prevalence of mental health help seeking among rural male adolescents in Australia should be sensitive to context-specific barriers and designed to meet their unique needs. Adolescent-focused digital interventions and strengthened family and community engagement are vital to ensuring equitable access to mental health services for adolescents in rural Australia.

**Supplementary Information:**

The online version contains supplementary material available at 10.1186/s13034-026-01022-7.

## Introduction

Poor mental health among children and adolescents is a major global health issue, with one in seven individuals experiencing a mental health disorder [1, 2]. This accounts for 15% of the global burden of disease in this age group [3]. Mental health conditions often develop early in life, with one-third of these conditions emerging before the age of 14 years and half before the age 18 [4]. Poor mental health among children and adolescents is also a pressing issue in Australia [5]. In 2020, nearly 27% of Australian adolescents (15–19 years) experienced psychological distress [1]. Despite the growing burden, only 22% of adolescents received support for their mental illness [6]. Approximately 25% of Australian adolescents aged 15–24 years reside in rural and remote areas [7]. These adolescents often face distinct structural and socioeconomic challenges that may influence their mental health and access to care. Key barriers include limited availability of mental health services, reduced educational and employment opportunities, higher levels of socioeconomic disadvantage, and geographic isolation [8]. These factors, however, may not uniformly increase vulnerability but contribute to a unique risk profile that differs from that of urban adolescents. Recognising this diversity is critical for developing context-sensitive approaches to adolescent mental health.

Mental health help-seeking behaviour is an adaptive coping strategy that involves seeking external support to address emotional or psychological issues and challenges. Adolescents’ mental health help-seeking behaviours are shaped by a complex interplay of personal (e.g., stigma, mental health literacy), social (e.g., family and peer support), and structural (e.g., service availability) factors [9]. One important influence is the availability of, and attitudes toward, different sources of support. These include formal support systems, including general practitioners, adolescent-friendly services such as headspace centres, and school counselling [1]. Adolescents also rely heavily on informal supports, including friends, parents, siblings, carers, and other trusted adults [10]. Online non-face-to-face support systems, including telephone helplines (e.g., Kids Helpline, Lifeline), and online platforms such as eheadspace and ReachOut [11] and social media platforms (e.g., TikTok, Facebook, Instagram) may also play a role in shaping help-seeking by offering information and peer validation [1]. Studies in Australia show that adolescents commonly seek help for emotional distress, depressive symptoms, anxiety, and self-harm, often turning first to informal sources [10]. These issues may prompt help-seeking when distress becomes difficult to manage, and informal pathways are often preferred because they feel more accessible, familiar, and less stigmatising.

Despite existing modern mental health support services, help-seeking rates remain low for rural adolescents in Australia [12]. Therefore, understanding adolescents’ mental health help-seeking behaviours in rural and remote Australia is vital for addressing the urban-rural disparities in mental health outcomes . While some studies have explored help-seeking behaviours of adolescents in rural Australia [13, 14], a current and comprehensive understanding of urban-rural disparities in adolescent mental health help-seeking is lacking. Furthermore, few studies have explored how varying levels of remoteness interact with factors that influence help-seeking of Australian adolescents. Examining how remoteness interacts with help-seeking of in this population will offer a more nuanced understanding of how geographic context may moderate the relationship between other determinants and help-seeking behaviour. Even within rural areas, varying degrees of remoteness may reflect differences in available resources and levels of economic development, which can shape the context of help-seeking behaviour. Thus, further examining variation across levels of remoteness could help clarify the specific factors influencing help-seeking.

Therefore, the aims of this study were to (i) understand the pattern of mental health help-seeking behaviours among Australian adolescents living in rural and remote areas, (ii) identify the factors associated with their help-seeking behaviours, and (iii) explore whether there are any urban-rural disparities in the strength of association between help-seeking behaviours and their associated factors. In this study, ‘mental health help-seeking behaviour’ refers to actions taken by adolescents to seek support for mental health concerns, including both professional sources (e.g., general practitioners, psychologists) and informal sources (e.g., family, friends, online forums). Where relevant, we distinguish between professional and informal help-seeking to clarify the nature of support accessed. The findings of this study will provide policymakers with invaluable insights needed to make evidence-based decisions when developing future programmes to enhance adolescent mental health in Australia.

## Methods

### Data sources

This study analysed data from the “Growing Up in Australia: The Longitudinal Study of Australian Children (LSAC)”. LSAC is the first nationally representative longitudinal cohort study of the life trajectories of children and their families across all states and territories in Australia. LSAC was initiated and funded by the Australian Government Department of Social Services (DSS) and conducted in collaboration with the Australian Institute of Family Studies (AIFS), the Australian Bureau of Statistics (ABS), and a consortium of leading researchers and experts from universities and research agencies. The LSAC study began in 2004 (Wave 1) with two groups of children: the B (“baby”) cohort, who were 0–1 year old at the start of the study, and the K (“kindergarten”) cohort, who were 4–5 years old at baseline [15]. Data have been collected every two years since then. Both in B- and K-cohort data were collected through multiple informants, including children, parents, carers and teachers [16]. Data were collected adopting various approaches including interview through face-to-face, computer-assisted, web-based and telephone, direct assessment and linking through linked administrative data. Help-seeking behaviours were self-reported by the study children with consent obtained from their parents or carers [16].

### LSAC study design

LSAC adopted a two-stage cluster sampling design. Children from both cohorts were selected from 311 postcodes across states and territories, with postcodes chosen based on probability proportional to size selection where possible, and with equal probability for small population postcodes. On average, 40 children per postcode were selected in larger states and 20 in smaller states and territories. The study follows two overlapping cohorts in a cross-sequential design, covering main Waves 1–8 from 2004 to 2018. The B-cohort began with 5,107 children (Wave 1) with information collected biannually at ages 2/3 years (Wave 2), 4/5 (Wave 3), 6/7 (Wave 4), 8/9 (Wave 5), 10/11 (Wave 6), 12/13 (Wave 7), and 14/15 years (Wave 8). Due to attrition, 3,127 children from the B-cohort and 3,037 children from the K-cohort remained by Wave 8. The detailed sampling design is described elsewhere [17].

### Selection of analytical sample for this study

We selected study participants from the B- and K-cohort of the latest available main Wave 8 of LSAC data. The initial sample included 3,127 participants from the B-cohort and 3,037 from the K-cohort. From the B-cohort, we excluded participants who reported no personal or emotional problems (*n* = 431) and those with missing data due to refusal to take part in the survey, inapplicability, skipped responses, and other reasons (*n* = 155). This exclusion of 431 participants was necessary because help-seeking questions were only asked of participants who reported experiencing any personal or emotional problems. Similarly, for the K-cohort, we excluded data for 741 participants (551 who had no personal or emotional problems and 190 for missing information). After these exclusions, the eligible sample comprised 2,541 participants from the B-cohort and 2,296 from the K-cohort. Combining these samples resulted in a final analytical sample of 4,837 participants (aged 14-19 years) for this study. We provided further details on the selection of the analytical sample in the supplementary section (Fig. S1).

### Measurements

*Outcome:* Mental help-seeking behaviour was the outcome of interest in this study. LSAC collects self-reported responses regarding whether adolescents sought help for emotional or personal problems within the 12 months preceding the survey completion. There were 13 help-seeking sources in the survey, which were further categorised into three primary groups in this study: formal, informal, and non-face-to-face (non-F2F) help-seeking [10]. *Formal help-seeking behaviour* was measured if the help-seeking sources were: (i) a teacher, (ii) other school staff, (iii) a family doctor or general practitioner (GP), or (iv) a mental health professional. Seven sources were defined as *informal help-seeking*: (i) a boyfriend, girlfriend or partner, (ii) a friend, (iii) a parent, (iv) a brother or sister, (v) another relative or family member, (vi) another adult, or (vii) someone else not listed before. Two other sources, (i) a phone helpline and (ii) the internet, were considered *non-F2F help-seeking* in this study. Adolescents who sought help from any of the 13 sources were classified as having engaged in *any help-seeking behaviour* (see further details in Table S1). Adolescents might have sought help from multiple sources; therefore, these groups are not mutually exclusive.

*Exposure of interest:* Remoteness was the exposure variable of interest. ABS measured the remoteness index under the construct of neighbourhood status within the housing topic, classifying it into five categories. We re-categorised participants into three groups based on their residence: major city, inner regional, and outer regional or remote area. According to the AIHW [8], rural and remote areas encompass all areas outside Australia’s major cities. Therefore, in this paper, rural areas refer broadly to rural and remote areas, which includes the remoteness categories of inner regional, outer regional, remote, and very remote, while urban areas refer to major cities. The supplementary section (Table S1) provides detailed information about the remoteness measurement.

*Predictors:* We included several predictors in this study based on the existing literature to understand the socio-demographic profile of the study participants and identify factors associated with help-seeking behaviours. A total of 24 predictor variables were assessed across six broad categories: (i) demographics of study participants (four variables), (ii) socio-economic status (three variables), (iii) health and well-being (four variables), (iv) lifestyle-related factors (six variables), (v) parental or family-related factors (three variables), and (vi) social and community-related factors ( four variables). The demographic variables included: age (14–15, 18–19 years), sex (female, male), main language spoken at home (English, Non-English), and Indigenous status ( Indigenous, Non-Indigenous). Socio-economic status variables comprised: primary carer’s financial hardship (yes, no), primary carer’s educational status (< 12 years, diploma/certificate, university degree), and Socio-Economic Indexes for Areas (SEIFA) (1st quantile: the most disadvantaged; 5th quantile: the least disadvantaged). Health and well-being factors included: general health condition (excellent, very good, good, fair or poor), ongoing anxiety or depression (yes, no), special health care needs (yes, no) and suicidal thoughts and behaviours (yes, no). The following lifestyle-related factors were considered: ever had an alcoholic drink (yes, no), ever smoked part of a cigarette (yes, no), ever tried any drugs (yes, no), physical activity levels (meet WHO recommendations, not meeting recommendations), screen time (none or < 2 h, ≥ 2 h per day), and social media exposure (none, 1 to 4 social networks, > 4 social networks). Parental or family-related factors included: single parenthood (yes, no), disability of any family members (yes, no), and relationship with parents (good, poor). Social and community-related factors included: bullying victimisation (yes, no), experiences of discrimination or unfair treatment (yes, no), involvement in community activities (yes, no), and participation in religious or spiritual groups (yes, no) (see Table S1 for further details on variables measurement and Table S2 for validation of predictors selection).

### Statistical analysis

We conducted descriptive analyses to outline the participants’ profiles, followed by bivariate analyses to examine the prevalence of help-seeking behaviours across all predictor variables. The weighted prevalence of help-seeking behaviours was estimated to understand the level of help-seeking behaviours among adolescents who experienced any mental health problems. Chi-square tests were used to examine differences in help-seeking behaviours across key demographic and contextual factors Table (S3). Disparities in the prevalence were also assessed across different demographic groups, such as age and sex, as well as by remoteness. To assess multicollinearity among predictor variables, we calculated the Variance Inflation Factor (VIF) for each independent variable. A VIF value above 5.0 was considered indicative of multicollinearity [18]. Where multicollinearity was detected, we removed the redundant predictor, and the model was further assessed for its stability and interpretability. We conducted bivariate and multivariate analyses between covariates and the outcome variables to identify the factors associated with help-seeking behaviours. Considering the study design (clustered at postcode) and the binary nature of the outcome measure (yes/no), we adopted a cluster-adjusted logistic regression model to examine the associations between outcome and predictor variables. The unadjusted estimates were derived from the bivariate model, analysing individual variables with the outcome variable. The adjusted estimates were generated from a model by mutually accounting for all covariates. The significant predictors from the adjusted model were further assessed in an interaction model (predictor × remoteness) individually in a separate model for each outcome to determine whether the strength of the association varied across remoteness categories. In this interaction analysis, the strength of association was compared between urban (major cities) vs. rural areas ( inner regional, outer regional or remote areas). We reported both adjusted and unadjusted odds ratio (ORs) with their corresponding 95% confidence intervals (CIs) as a measure of the strength of association. We performed all analyses using STATA 18.0 (StataCorp, College Station, TX, USA).

**Fig. 1:**
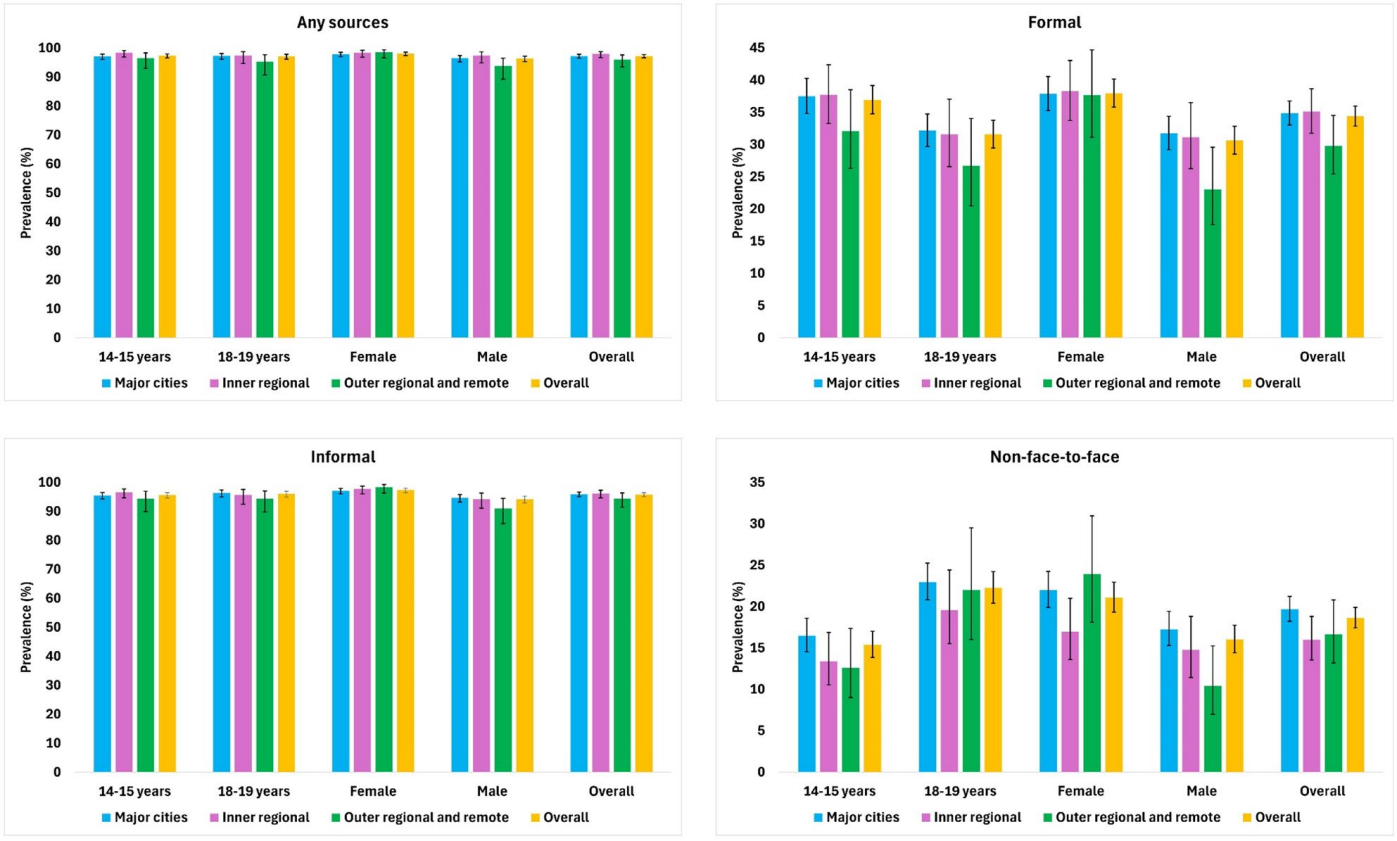
Prevalence of mental health help-seeking behaviours of adolescents in Australia, by age, sex, and remoteness (weighted). Error bars represent 95% confidence intervals (CI)

### Missing data imputation

There were some missing data on covariates such as main language spoken at home, primary carer’s financial hardship, primary carer’s educational status, special health care needs, and physical activity (Table S1). Missing data on covariates were addressed using multiple imputation (MI) techniques [19]. MI involves estimating a set of plausible values for missing data by utilising the distribution of observed data to create a ‘complete’ dataset. Chained equations were implemented to accommodate different variable types (e.g., binary, ordinal, continuous), employing the STATA command ‘*mi impute chained*’. A chained equation is a sequential univariate imputation method to specify prediction equations conditionally. This imputation process encompassed 1,000 iterations, resulting in the generation of 30 imputed datasets. Imputation validity was verified by comparing covariate distributions before and after the imputation. Imputation accuracy was evaluated through parameters such as the relative increase in variance, fraction of missing information, degrees of freedom, relative efficiency, and between-imputation and within-imputation variance estimates. Regression analyses were conducted using both available and imputed complete datasets. No substantial differences were observed in the estimates between the imputed dataset and the available cases. Hence, the reported estimates are derived from the imputed dataset.

## Results

Table 1 shows the characteristics of the study participants. About half (52%) of the participants were female, 52% were adolescents aged 14–15 years, and the vast majority (98%) identified as non-Indigenous (Table 1). Moreover, around 14% of adolescents had primary carers who experienced financial hardship in meeting family expenses. Over two-thirds of the adolescents resided in urban areas, with the remaining (31%) living in rural and remote communities (Table 1).

Figure 1 demonstrates the prevalence of mental health help-seeking behaviours of adolescents in Australia, by age, sex, and remoteness. Overall, informal help-seeking was the most prevalent help-seeking behaviour (95.84%, 95% CI: 95.12–96.46), whereas formal (34.42%, 95% CI: 32.90–35.98) and non-F2F help-seeking (18.64%, 95% CI: 17.44–19.91) were less prevalent (Fig. 1). Adolescents in rural areas consistently showed lower prevalence of formal, informal, and non-F2F help-seeking compared to their urban counterparts, although most differences were not statistically significant. Female adolescents exhibited higher help-seeking behaviour across all sources and remoteness categories compared to their male counterparts (Fig. 1). For example, male adolescents in remote areas had significantly lower prevalence of non-F2F help-seeking (10.43%, 95% CI: 7.00–15.27) compared to their female counterparts (23.96%, 95% CI: 18.12–30.98).

**Table 1:** Characteristics of the study participants (*N* = 4,837)

Participants’ characteristics	% (*n*)
Age	
- 14–15 years	52.53 (2,541)
- 18–19 years	47.47 (2,296)
Sex	
- Female	51.95 (2,513)
- Male	48.05 (2,324)
Main language spoken at home	
- English	90.72 (4,388)
- Non-English	6.39 (309)
- Missing	2.89 (140)
Indigenous status	
- Non-Indigenous	97.77 (4,729)
- Indigenous	2.23 (108)
Primary carer’s financial hardship	
- No	79.43 (3,842)
- Yes	13.75 (665)
- Missing	6.82 (330)
Primary carer’s education	
- < 12 years	12.34 (597)
- Diploma/certificate	47.53 (2,299)
- University degree	28.01 (1,355)
- Missing	12.11 (586)
SEIFA	
- First quantile (q1, most disadvantaged)	13.07 (632)
- Second quantile (q2)	17.86 (864)
- Third quantile (q3)	19.31 (934)
- Fourth quantile (q4)	23.09 (1,117)
- Fifth quantile (q5, least disadvantaged)	26.63 (1,288)
- Missing	0.04 (2)
Remoteness	
- Major cities	68.51 (3,314)
- Inner regional	20.67 (1,000)
- Outer regional or remote	10.77 (521)
- Missing	0.04 (2)

SEIFA = Socio-Economic Index for Areas

With regard to specific help-seeking sources, friends (80.00%, 95% CI: 78.64–81.30) served as the most common informal support network, followed by parents (73.78%, 95% CI: 72.29–75.22). The internet (17.19%, 95% CI: 16.03–18.41) was the most common source of help sought among non-F2F help-seeking sources. For formal help-seeking sources, teachers (20.01%, 95% CI: 18.75–21.33) were the most frequently approached. Focusing on professional help-seeking, notable disparities were observed: it was significantly less common among adolescents in remote areas (7.72%, 95% CI: 5.39–10.93) compared to those in urban areas (12.20%, 95% CI: 10.97–13.54). Within remote areas, male adolescents reported a much lower prevalence of professional help-seeking (2.76%, 95% CI: 1.33–5.63) than their female peers (13.53%, 95% CI: 9.08–19.70) (Table S3). We have provided further details on specific help-seeking sources in supplementary Table S3.

Table 2  presents the prevalence for formal and overall (any) help-seeking sources, while supplementary Table S4 reports prevalence for informal and non-F2F help-seeking sources, categorised by exposure variables and predictors. We found several factors that were significantly associated with help-seeking behaviours in this study. Male adolescents showed lower odds of seeking formal help (adjusted OR 0.80, 95% CI: 0.69–0.94). In contrast, however, adolescents with ongoing anxiety or depression, special healthcare needs, suicidal thoughts and behaviours, single-parent family backgrounds, good relationships with their parents, those who experienced bullying, unfair treatment, or discrimination, or who participated in community groups had significantly higher odds of seeking formal help (Table 3). Male adolescents were also less likely to seek informal help. Conversely, those with ongoing anxiety or depression, who had tried drugs, had good relationships with their parents, experienced bullying, or participated in community groups showed greater odds of informal help-seeking. Additionally, older adolescents (18–19 years), those living in the least disadvantaged areas, those with ongoing anxiety or depression, those who had suicidal thoughts or behaviours, had exposure to more than four social networks, or those who experienced discrimination or unfair treatment demonstrated higher odds of seeking non-F2F help. Notably, adolescents with good relationships with their parents had lower odds of seeking non-F2F help (Table S5).

**Table 2:** Prevalence of adolescents’ help-seeking behaviours from formal and any sources, by predictors

Factors/Predictors	Formal			Any sources	
	N	% (*n*)	*p*-value	% (*n*)	*p*-value
*Demographics*					
Age					
- 14–15 years	2541	36.60 (930)	0.007	97.28 (2,472)	0.545
- 18–19 years	2296	32.93 (756)		97.56 (2,240)	
Sex					
- Female	2513	38.88 (977)	< 0.001	98.05 (2,464)	0.004
- Male	2324	30.51 (709)		96.73 (2,248)	
Main language spoken at home					
- English	4388	35.05 (1,538)	0.620	97.52 (4,279)	0.909
- Non-English	309	33.66 (104)		97.41 (301)	
- Missing	140	31.43 (44)		94.29 (132)	
Indigenous status					
- Non-Indigenous	4729	34.81 (1,646)	0.631	97.42 (4,607)	0.898
- Indigenous	108	37.04 (40)		97.22 (105)	
*Socio-economic status*					
Primary carer’s financial hardship					
- No	3842	35.03 (1,346)	0.549	97.50 (3,746)	0.752
- Yes	665	33.83 (225)		97.29 (647)	
- Missing	330	34.85 (115)		96.67 (319)	
Primary carer’s education					
- < 12 years	597	34.51 (206)	0.674	97.32 (581)	0.781
- Diploma/certificate	2299	34.75 (799)		97.26 (2,236)	
- University degree	1355	36.09 (489)		97.64 (1,323)	
- Missing	586	32.76 (192)		97.61 (572)	
SEIFA					
- First quantile (q1, most disadvantaged)	632	32.91 (208)	0.092	96.68 (611)	0.576
- Second quantile (q2)	864	36.23 (313)		97.57 (843)	
- Third quantile (q3)	934	36.19 (338)		97.86 (914)	
- Fourth quantile (q4)	1117	31.96 (357)		97.67 (1,091)	
- Fifth quantile (q5, least disadvantaged)	1288	36.41 (469)		97.13 (1,251)	
- Missing	2	50.00 (1)		100.00 (2)	
Remoteness					
- Major cities	3314	35.55 (1,178)	0.156	97.37 (3,227)	0.121
- Inner regional	1000	34.40 (344)		98.10 (981)	
- Outer regional or remote	521	31.29 (163)		96.35 (502)	
- Missing	2	50.00 (1)		100.00 (2)	
*Health and well-being*					
General health condition					
- Excellent	893	32.92 (294)	< 0.001	97.42 (870)	< 0.001
- Very good	2017	33.52 (676)		98.02 (1,977)	
- Good	1415	34.49 (488)		97.39 (1,378)	
- Fair or poor	463	43.41 (201)		94.60 (438)	
- Missing	49	55.10 (27)		100.00 (49)	
Has ongoing anxiety or depression					
- No	4061	30.56 (1,241)	< 0.001	97.22 (3,948)	0.043
- Yes	736	59.51 (438)		98.51 (725)	
- Missing	40	17.50 (7)		97.50 (39)	
Special health care needs					
- No	3845	31.50 (1,211)	< 0.001	97.27 (3,740)	0.219
- Yes	854	48.13 (411)		98.01 (837)	
- Missing	138	46.38 (64)		97.83 (135)	
Suicidal thoughts and behaviours					
- No	3712	31.06 (1,153)	< 0.001	97.58 (3,622)	0.129
- Yes	1072	48.51 (520)		96.74 (1,037)	
- Missing	53	24.53 (13)		100.00 (53)	
*Lifestyle-related factors*					
Ever had alcoholic drink					
- No	1328	36.82 (489)	0.079	97.29 (1,292)	0.746
- Yes	3458	34.12 (1,180)		97.46 (3,370)	
- Missing	51	33.33 (17)		98.04 (50)	
Ever smoked part of a cigarette					
- No	3493	35.64 (1,245)	0.056	97.22 (3,396)	0.204
- Yes	1318	32.70 (431)		97.88 (1,290)	
- Missing	26	38.46 (10)		100.00 (26)	
Ever tried any drug					
- No	3615	35.44 (1,281)	0.134	97.15 (3,512)	0.055
- Yes	1201	33.06 (397)		98.17 (1,179)	
- Missing	21	38.10 (8)		100.00 (21)	
Physical activity (Vigorous- intensity)					
- Does not meet WHO recommendations	3369	34.67 (1,168)	0.061	97.51 (3,285)	0.894
- Meet WHO recommendations	1074	31.56 (339)		97.58 (1,048)	
- Missing	394	45.43 (179)		96.19 (379)	
Screen time (per day)					
- No or < 2 h	2860	35.63 (1,019)	0.336	97.73 (2,795)	0.105
- ≥ 2 h	1914	34.27 (656)		96.97 (1,856)	
- Missing	63	17.46 (11)		96.83 (61)	
Social media exposure					
- None	109	41.28 (45)	< 0.001	96.33 (105)	0.409
- 1–4 social networks	3585	33.28 (1,193)		97.27 (3,487)	
- > 4 social networks	1092	40.38 (441)		97.89 (1,069)	
- Missing	51	13.73 (7)		100.00 (51)	
*Parent/family-related factors*					
Single parenthood					
- No	3850	34.13 (1,314)	0.019	97.53 (3,755)	0.267
- Yes	967	38.16 (369)		96.90 (937)	
- Missing	20	15.00 (3)		100.00 (20)	
Family member with a disability (anyone)					
- No	2886	33.06 (954)	< 0.001	97.61 (2,817)	0.264
- Yes	1922	37.88 (728)		97.09 (1,866)	
- Missing	29	13.79 (4)		100.00 (29)	
Relationship with parents					
- Poor (bottom 25%)	1348	36.80 (496)	0.019	94.58 (1,275)	< 0.001
- Good (top 75%)	3442	34.34 (1,182)		98.49 (3,390)	
- Missing	47	17.02 (8)		100.00 (47)	
*Social/community-related factors*					
Bullying victimisation					
- No	2858	31.18 (891)	< 0.001	97.03 (2,773)	0.037
- Yes	1903	40.78 (776)		98.00 (1,865)	
- Missing	76	25.00 (19)		97.37 (74)	
Discriminated/unfair treatment					
- No	3343	30.30 (1,013)	< 0.001	97.25 (3,251)	0.344
- Yes	1449	45.82 (664)		97.72 (1,416)	
- Missing	45	20.00 (9)		100.00 (45)	
Participation in community groups					
- No	2413	31.45 (759)	< 0.001	96.85 (2,337)	0.017
- Yes	2387	38.33 (915)		97.95 (2,338)	
- Missing	37	32.43 (12)		100.00 (37)	
Active in religious or spiritual groups					
- No	4074	33.87 (1,380)	0.003	97.25 (3,962)	0.139
- Yes	723	39.56 (286)		98.20 (710)	
- Missing	40	50.00 (20)		100.00 (40)	

SEIFA = Socio-Economic Index for Areas, WHO = World Health Organization

**Table 3:** Factors associated with formal and any help-seeking behaviours among adolescents in Australia (*N* = 4,837)

Factors/Predictors	Formal; OR (95% CI)		Any sources; OR (95% CI)	
	Unadjusted	Adjusted (95% CI)	Unadjusted	Adjusted (95% CI)
**Age**				
- 18–19 years	0.85 (0.76–0.96)*	0.85 (0.71–1.02)	1.12 (0.78–1.60)	1.28 (0.75–2.19)
- Ref: 14–15 years				
**Sex**				
- Male	0.69 (0.61–0.78)*	0.80 (0.69–0.94)*	0.59 (0.41–0.85)*	0.68 (0.45–1.03)
- Ref: Female				
**Main language spoken at home**				
- Non-English	0.94 (0.74–1.20)	0.97 (0.72–1.31)	0.96 (0.46–1.98)	1.07 (0.51–2.26)
- Ref: English				
**Indigenous status**				
- Indigenous	1.10 (0.74–1.64)	1.33 (0.86–2.06)	0.93 (0.29–2.96)	0.76 (0.22–2.72)
- Ref: Non-Indigenous				
**Primary carer’s financial hardship**				
- Yes	0.96 (0.81–1.14)	0.82 (0.67–0.99)*	0.96 (0.58–1.59)	1.08 (0.60–1.96)
- Ref: No				
**Primary carer’s education**				
- Diploma/certificate	1.03 (0.85–1.24)	0.95 (0.77–1.17)	1.00 (0.58–1.73)	0.91 (0.48–1.72)
- University degree	1.09 (0.89–1.32)	0.99 (0.78–1.25)	1.14 (0.63–2.07)	1.01 (0.49–2.08)
- Ref: <12 years				
**SEIFA**				
- Second quantile (q2)	1.16 (0.93–1.44)	1.24 (0.95–1.60)	1.38 (0.75–2.55)	1.38 (0.68–2.79)
- Third quantile (q3)	1.16 (0.93–1.43)	1.21 (0.96–1.54)	1.57 (0.84–2.92)	1.50 (0.75–2.98)
- Fourth quantile (q4)	0.96 (0.78–1.18)	0.96 (0.74–1.24)	1.44 (0.80–2.59)	1.54 (0.77–3.09)
- Fifth quantile (q5, least disadvantaged)	1.17 (0.96–1.43)	1.20 (0.92–1.56)	1.16 (0.67–2.00)	0.86 (0.46–1.62)
- Ref: First quantile (q1, most disadvantaged)				
**Remoteness**				
- Inner regional	0.95 (0.82–1.10)	0.93 (0.78–1.10)	1.39 (0.84–2.30)	1.55 (0.86–2.81)
- Outer regional or remote	0.83 (0.68–1.01)	0.83 (0.65–1.05)	0.71 (0.43–1.18)	0.67 (0.33–1.38)
- Ref: Major cities				
**General health condition**				
- Very good	1.03 (0.87–1.22)	0.96 (0.80–1.16)	1.31 (0.78–2.20)	1.46 (0.81–2.64)
- Good	1.08 (0.91–1.29)	0.86 (0.70–1.07)	0.99 (0.58–1.68)	1.25 (0.67–2.36)
- Fair or poor	1.57 (1.25–1.98)*	1.06 (0.80–1.41)	0.47 (0.26–0.83)*	0.63 (0.28–1.42)
- Ref: Excellent				
**Has ongoing anxiety or depression**				
- Yes	3.30 (2.81–3.88)*	2.39 (1.97–2.90)*	1.90 (1.02–3.55)*	2.28 (0.99–5.29)
- Ref: No				
**Special health care needs**				
- Yes	2.01 (1.73–2.34)*	1.21 (1.01–1.46)*	1.35 (0.80–2.27)	1.10 (0.63–1.91)
- Ref: No				
**Suicidal thoughts and behaviours**				
- Yes	2.08 (1.81–2.38)*	1.56 (1.31–1.85)*	0.74 (0.50–1.10)	1.01 (0.64–1.60)
- Ref: No				
**Ever had alcoholic drink**				
- Yes	0.89 (0.78–1.01)	0.91 (0.78–1.06)	1.05 (0.71–1.56)	0.83 (0.49–1.41)
- Ref: No				
**Ever smoked part of a cigarette**				
- Yes	0.88 (0.77–1.01)	0.83 (0.67–1.02)	1.32 (0.86–2.02)	0.99 (0.51–1.91)
- Ref: No				
**Ever tried any drug**				
- Yes	0.90 (0.79–1.04)	0.95 (0.78–1.16)	1.58 (0.99–2.51)	1.94 (0.89–4.22)
- Ref: No				
**Physical activity (moderate to vigorous-intensity)**				
- Meet WHO recommendations	0.87 (0.75–1.01)	0.93 (0.79–1.10)	1.03 (0.66–1.61)	1.11 (0.66–1.87)
- Ref: Does not meet WHO recommendations				
**Screen time (per day)**				
- ≥ 2 h	0.94 (0.83–1.06)	1.01 (0.88–1.17)	0.74 (0.51–1.05)	1.02 (0.65–1.59)
- Ref: < 2 h				
**Social media exposure**				
- 1–4 social networks	0.71 (0.48–1.05)	0.82 (0.55–1.23)	1.36 (0.49–3.77)	1.90 (0.60–6.00)
- > 4 social networks	0.96 (0.64–1.43)	1.04 (0.69–1.58)	1.78 (0.60–5.25)	2.61 (0.75–9.09)
- Ref: None				
**Single parenthood**				
- Yes	1.19 (1.03–1.38)*	1.21 (1.02–1.43)*	0.79 (0.52–1.20)	0.63 (0.40–0.97)*
- Ref: No				
**Family member with a disability (anyone)**				
- Yes	1.23 (1.09–1.39)*	1.14 (0.99–1.32)	0.82 (0.57–1.17)	0.73 (0.49–1.08)
- Ref: No				
**Relationship with parents**				
- Good (top 75%)	0.90 (0.79–1.02)	1.26 (1.08–1.48)*	3.72 (2.59–5.34)*	4.68 (2.98–7.34)*
- Ref: Poor (bottom 25%)				
**Bullying victimisation**				
- Yes	1.51 (1.34–1.70)*	1.35 (1.16–1.57)*	1.51 (1.03–2.22)*	1.83 (1.13–2.96)*
- Ref: No				
**Discrimination/unfair treatment**				
- Yes	1.94 (1.71–2.20)*	1.41 (1.21–1.65)*	1.21 (0.81–1.81)	1.39 (0.85–2.29)
- Ref: No				
**Participation in community groups**				
- Yes	1.35 (1.20–1.52)*	1.34 (1.15–1.57)*	1.55 (1.08–2.23)*	1.47 (0.93–2.31)
- Ref: No				
**Active in religious or spiritual groups**				
- Yes	1.27 (1.08–1.50)*	1.17 (0.98–1.40)	1.54 (0.86–2.75)	1.80 (0.92–3.54)
- Ref: No				

CI = confidence interval, OR = odds ratio, SEIFA = Socio-Economic Index for Areas, WHO = World Health Organization

*p < 0.05

Despite identifying several significant factors associated with help-seeking behaviours, the strength of these associations did not differ statistically between rural and urban areas, except for community participation in informal help-seeking and financial hardship in formal help-seeking (Table S6). Our interaction model showed that the strength of the association between community participation and informal help-seeking behaviours varied by remoteness (urban vs. rural). Specifically, community participation (e.g., engagement in community/welfare, emergency services, adolescent/mentoring, cultural, animal welfare, environment, immigrant/refugee assistance, international aid/development, human rights, professional associations, ethnic societies) had a significantly lower effect on seeking informal help in rural areas than urban areas (adjusted OR: 0.37, 95% CI: 0.14–0.97 for community participants in outer regional and remote areas). Furthermore, the association between financial hardship and help-seeking differed across geographic areas. Rural adolescents experiencing financial hardship had higher odds of seeking help than their urban counterparts (adjusted OR: 1.70, 95% CI: 1.05–2.78 for financial hardship in inner regional areas) (Table S6).

## Discussion

In this study, using a nationally representative sample of Australian adolescents, we examined the prevalence of help-seeking behaviours across four broader categories: formal, informal, non-F2F, and any sources, stratified by age, gender, and geographic remoteness. We also identified key factors associated with help-seeking behaviours and explored how remoteness moderates the strengths of these associations. Across all demographic groups and remoteness categories, there was high help-seeking behaviour for informal sources of support, while the prevalence of non-F2F and formal sources of support were significantly lower than informal sources of support, particularly in outer regional and remote areas. Adolescents in outer regional and remote areas had a significantly lower prevalence of formal help-seeking from mental health professionals compared to their urban peers, with males in particular exhibiting substantially lower prevalence than females. While several factors were associated with different forms of help-seeking (including mental health conditions, parent-child relationships, single-parent family backgrounds and participation in community activities), ongoing anxiety or depression and parent-child relationships were consistently associated with all types of help-seeking behaviours. Geographic variations were observed in the effects of community participation on informal help-seeking and financial hardship on formal help-seeking. Our findings highlight the influence of demographic, social and geographical factors on adolescents’ help-seeking behaviours, revealing targets to inform the development of equitable interventions to improve help-seeking for adolescents in Australia.

The significantly lower prevalence of formal and non-F2F help-seeking compared to informal support highlights the importance of personal networks for mental health support among Australian adolescents. These findings are consistent with existing evidence, which emphasises the preference for informal sources like family and friends among young people over professional services [20]. In alignment with prior research, a tendency for self-reliance and negative perceptions of professional help likely pose significant barriers to help-seeking among adolescents in Australia [21]. The lower prevalence of formal and non-F2F help-seeking among rural adolescents, compared to their urban counterparts, highlights critical geographical disparities in perceived or actual barriers to accessing mental health support, suggesting an urgent need for targeted interventions [22, 23]. Adolescents in outer regional and remote areas exhibited a significantly lower prevalence of seeking formal support from mental health professionals compared to those living in urban settings. This disparity, and possibly the underutilisation of formal mental health services (family doctors/GPs, mental health professionals, school teachers and other staff) may reflect lower awareness of, or trust in, available resources and systemic inequities in the availability and accessibility of mental health services in rural and remote regions, where access to professional and structured services remains limited [14, 24]. The reduced engagement with mental health professionals in these areas may contribute to unmet mental health needs for young people, requiring innovative solutions to address geographical barriers associated with help-seeking, such as telehealth initiatives and community-based mental health programs tailored to the unique needs of rural adolescents. The Federal Government is offering incentives to attract mental health professionals to rural areas for better mental health support [25].

We also found that fewer male adolescents sought help from both formal and non-F2F sources than females. This disparity was particularly pronounced in regional and remote areas, where fewer males sought support from mental health professionals. There are a number of reasons why adolescent males may seek out help less frequently compared to adolescent females, including masculinity norms, such as social expectations of self-reliance and emotional restraint, as well as gendered socialisation processes [26, 27], which may discourage males from seeking help for a range of issues, including mental health problems. In addition, males more often perceive themselves as self-confident and capable of handling mental health challenges independently [28]. In regional and remote areas, a tight-knit community may further perpetuate mental health stigma among male adolescents, leading to concerns about confidentiality and fear of judgment [29]. Young males may hesitate to seek professional help due to concerns about confidentiality and fear of negative peer perceptions if their diagnosis becomes known [24]. Our findings suggest a need for gender-focused mental health programs, particularly for male adolescents in regional and remote communities. Implementing peer support initiatives and male-focused campaigns can help overcome barriers to help-seeking. Although research assessing effectiveness is needed, adolescent mental health programs should focus on increasing awareness of available supports, improving recognition of mental health challenges, promoting self-help strategies and addressing stigma and stereotypes that negatively impact young people’s mental wellbeing.

Both ongoing anxiety or depression and good parent-child relationships were consistent predictors across all help-seeking categories. Adolescents experiencing persistent psychological challenges such as anxiety and/or depression may feel a stronger need and motivation for assistance, leading them to seek help. They may have increased awareness of mental health issues, access to services, and encouragement for help from different sources [30]. National initiatives such as Beyond Blue’s Australian National Depression Initiative have likely contributed to this trend by promoting mental health education, reducing stigma, and fostering engagement with professional support systems [31].

The association between good parental relationships and increased help-seeking behaviours underscores the importance of family dynamics in facilitating mental health support [32]. However, the lower odds of non-F2F help-seeking among adolescents with good parental relationships may suggest a preference for direct, in-person support within familial contexts. In this study, adolescents from single-parent families exhibited lower odds of any help seeking, which may reflect differential access to support systems and economic resources. Parents play an important role in helping adolescents seek support for mental health issues. They assist in identifying whether the problem is psychological, in finding appropriate help, and in ensuring access to services. Strong parent-child relationships can make it easier for parents to guide their children toward professional support and may sometimes reduce the need for such help by offering adequate emotional support [32]. Our findings underscore the important role of informal supports, particularly those provided by parents, friends, and peers, in shaping adolescents’ mental health help-seeking behaviours. This aligns with a substantial body of literature demonstrating that close relationships can facilitate help-seeking by offering emotional validation, encouragement, and practical assistance in navigating services [9, 33–35]. However, these relationships can also serve as barriers when characterised by stigma, limited mental health literacy, or dismissive attitudes (e.g., mental health problems are insufficient to warrant treatment) [36, 37]. While our results reaffirm the well-established influence of parental support, they also highlight the complexity of this dynamic: parental involvement can both enable and inhibit help-seeking, depending on the quality of communication and the attitudes held within the family. This dual role underscores the need for family-based interventions that not only empower but also educate caregivers to foster supportive environments. In rural contexts, where access to formal services may be constrained, informal support networks often become the primary source of help, making the engagement of families and peer groups especially critical in mental health promotion and intervention strategies. Overall, our findings support the influence of supportive social networks and social capital on mental health service utilisation [21].

The interaction effects observed in our study provide additional insights into how specific factors influence help-seeking behaviours across different remoteness categories. For instance, we found a lower effect of community participation on informal help-seeking for rural adolescents compared to their urban peers. In rural areas, participation in community activities may not facilitate informal help-seeking due to factors such as strong expectations of self-reliance and resilience, concerns about self-, social-and structural stigma surrounding mental health within tight-knit communities and a preference for maintaining privacy [38, 39]. While urban adolescent may find that engaging in community activities broadens their access to informal support networks, rural adolescents might feel constrained by local social norms, limiting their willingness to discuss mental health concerns within their community circles [38]. These findings suggest that community participation, including school-based wellbeing initiatives, peer support networks, and cross-sector partnerships between local services, schools, and families, may foster social cohesion [40]. However, it may not necessarily translate to increased informal help-seeking among rural adolescents. Our findings, therefore, suggest that the social mechanisms linking community participation to help-seeking differ by geographic context. Rather than assuming a uniform effect, interventions should be tailored to local norms, resources, and relational dynamics. In rural settings, strategies may need to go beyond stigma reduction and focus on building trust, safeguarding confidentiality, and adapting peer-based support to align with community values. This could include targeted educational initiatives, cross-sector partnerships, and efforts to strengthen informal networks, such as family, friends, and community groups, as accessible sources of emotional support. While remoteness was not independently associated with help-seeking in our models, this finding should be interpreted within a broader ecological framework. Geography intersects with service availability, transportation infrastructure, and community norms [41]. Our findings suggest that remoteness alone may not capture the full complexity of access barriers or supports.

The interaction between geography and financial hardship revealed that rural adolescents experiencing financial hardship had higher odds of seeking help than their urban counterparts, which indicates that socio-contextual factors shape help-seeking behaviours. Qualitative evidence from previous research suggests this pattern may reflect weaker social and financial support networks (e.g., help from extended family) and fewer employment opportunities in rural areas [42, 43], amplifying the stress of financial hardship and prompting greater reliance on help seeking. Additionally, rural adolescents may conceptualise help-seeking as a response to addressing material needs rather than mental health concerns, potentially mitigating stigma-related barriers. While these interpretations remain speculative, further research is needed to understand the mechanisms underlying this association to explain why rural adolescents experiencing financial hardship may be more likely to seek help than their urban peers.

The strength of our study lies in the use of representative data on children and adolescents in Australia. LSAC provided rich data on help-seeking behaviours and their associated factors, covering a wide range of topics. This enabled us to analyse help-seeking from individual, familial, and societal perspectives. A novel aspect of our study was the exploration of urban-rural disparities in help-seeking behaviours among Australian adolescents. In particular, we examined how remoteness interacts with factors that influence help-seeking among this population. We also disaggregated our prevalence estimates by age, gender, and other factors pertinent to adolescent help-seeking behaviours, as well as by specific sources of help-seeking. This approach offers valuable descriptive information that can inform future prevention and intervention programs for vulnerable groups. Additionally, the use of sample weights in our analyses ensured that the findings were more reflective of the original design and sample of Australian children and their families.

There were also several limitations. The mental health related variables we used in our study are self-reported, which may have introduced social desirability bias and recall inaccuracies. Missing values might have occurred due to non-response cases in web-based forms, not applicable or do not know, refusal or lack of answers, and for any skipped or unknown answers. To address the issue of missing data, we applied multiple imputation methods to estimate plausible values based on observed data patterns. While this approach helped reduce bias and improve the robustness of our analyses, it may not entirely eliminate the possibility of residual biases, as the accuracy of the imputations depends on the underlying assumptions and the quality of the available data. We assessed multicollinearity among the potential factors associated with help-seeking; however, it may not have been fully eliminated. As we used cross-sectional data, our findings identify potential associations but cannot establish causal relationships. Our study sample may not fully represent the diversity of Australia’s population, with underrepresented adolescent populations such as Indigenous communities, culturally and linguistically diverse (CALD) groups, and communities in extremely remote areas. While LSAC includes participants from diverse regions, its ability to address urban-rural disparities or remote populations may be limited by sample size constraints. Another limitation of the data presentation is that many of the 13 help-seeking sources in the dataset were grouped into three broad categories: formal, informal, and non-F2F. These broad categories may not be mutually exclusive, as participants could access multiple types of support (e.g., both formal and informal health services) simultaneously. In a study that considers multiple co-occurring help-seeking sources, more distinct patterns of combined use could be captured using person-centred approaches, such as latent class analysis [44, 45]. While this study focused on broad categories of help-seeking behaviours by geographic group, future studies could explore more granular patterns of help-seeking sources, both individual and combined, and examine how these configurations vary across geographic regions.

Addressing geographical and logistical barriers, especially in regional and rural settings, will require innovative solutions that actively involve young people from the respective communities. A key objective of the WHO Comprehensive Mental Health Action Plan 2013–2030 is to “provide comprehensive, integrated and responsive mental health and social care in community-based settings” [46]. The findings of this study could contribute to implementing the WHO action plan by informing planning approaches that support mental health help-seeking among adolescents in rural areas. In Australia, adolescent mental health is prominently addressed within broader national frameworks, including the National Action Plan for the Health of Children and Young People 2020–2030 [47] and the National Mental Health and Suicide Prevention Plan [48]. These initiatives focus on enhancing mental health outcomes for young people by prioritising community-based care and early intervention strategies. Additionally, they aim to overcome barriers faced in rural and remote areas, ensuring equitable access to mental health resources and services across diverse geographic regions. Therefore, in alignment with national and global objectives, to address disparities in mental health help-seeking among adolescents, particularly males in rural and remote Australian communities, policy initiatives must focus on equitable approaches to encouraging help seeking for professional support services and addressing the structural barriers limiting engagement.

Targeted strategies should integrate peer-based support models, reinforce informal networks and actively reduce stigma surrounding mental health problems. Additionally, policy frameworks should incorporate gender-sensitive approaches that specifically address the influence of social masculinity, encouraging help-seeking behaviours by challenging traditional norms of self-reliance and emotional restraint. While often framed as a barrier, self-reliance may also reflect adaptive coping strategies, developmental autonomy, or cultural values that prioritise independence [49]. Rather than viewing self-reliance as an obstacle to be overcome, interventions might instead leverage this trait by promoting self-guided help-seeking tools or peer-supported pathways.

Future research should explore the effectiveness of culturally and geographically tailored interventions, assessing their impact on adolescent mental health outcomes. Longitudinal studies examining the intersection of masculinity, rural identity, and mental health literacy could provide further insights into how these factors shape help-seeking behaviours, informing evidence-based policies that enhance adolescent mental health care accessibility and engagement. However, it is important to avoid homogenising rural communities. While some rural areas face significant isolation, others may benefit from strong social cohesion and local resourcefulness [50]. This diversity underscores the need for place-based approaches that recognise both the challenges and strengths of rural settings. To ensure relevance and sustainability, future mental health initiatives targeting rural adolescents should actively involve young people in their design and delivery. Co-design and participatory approaches, such as adolescent-led workshops, peer consultation, and patient and public involvement, have been shown to enhance engagement, cultural sensitivity, and service uptake [51]. For example, tailored educational campaigns and stigma-reduction programs are more likely to succeed when informed by the lived experiences of rural adolescent and grounded in community values, as demonstrated by the Live4Life model implemented across regional Australia [52]. Future interventions addressing adolescent mental health help-seeking behaviour, or more broadly adolescent mental health, should be context-sensitive and developed collaboratively with local stakeholders to avoid reinforcing existing barriers. Embedding adolescent voices in program development is not only ethically sound but also critical for fostering trust and sustainable impact.

## Supplementary Information

Supplementary Material.

## Data Availability

The datasets used in this study are available from the Australian Data Archive of the Australian Government DSS. De-identified datasets may be made available, subject to compliance with data access conditions and DSS approval.
