# Supplementary material for: Adolescent mental health help-seeking behaviours in rural Australia: cross-sectional analysis of a nationwide cohort study
Source: Child Adolesc Psychiatry Ment Health. 2026 Feb 4;20:34. doi: 10.1186/s13034-026-01022-7 (PMC12964690; doi:10.1186/s13034-026-01022-7)
Supplement: Supplementary file 1 — Supplementary Material. [file 13034_2026_1022_MOESM1_ESM.docx]

**Supplementary materials**

Selected study participants from Longitudinal Study of Australian Children (LSAC) B-cohort (wave 8), N = 3,127

Excluded participants:

- do not have a personal or emotional problem (N = 551)

- missing cases (N = 190)

Included participants:

- B-cohort (N = 2,541)

- K-cohort (N = 2,296)

Final analytical sample:

N = 4,837

Selected study participants from Longitudinal Study of Australian Children (LSAC) K-cohort (wave 8), N = 3,037

Excluded participants:

- do not have a personal or emotional problem (N = 431) - missing cases (N = 155)

**Fig. S1** Flowchart of analytical sample selection

**Table S1** Description of variables/indicators with their measurement used in this study

| **Variables/Indicators** | **Description** | **Measurement used in this study** | **Missing on covariates**  **n (%)** |
| --- | --- | --- | --- |
|  |  |  |  |
| ***Outcomes*** |  |  |  |
| Formal help-seeking | Formal help-seeking behaviour refers to instances in which study participants sought help for personal or emotional problems in the past 12 months from any of the following sources: a teacher, other school staff, a family doctor/GP, or a mental health professional (four items). For example, participants were asked, “Have you sought help for personal or emotional problems from any of these in the last 12 months?” Responses indicating help-seeking from a teacher, other school staff, family doctor/GP, or mental health professional were coded as “Yes” and categorised as formal help-seeking. | 1 = Yes, 0 = No | -- |
| Non-F2F help-seeking | Non-face-to-face (non-F2F) help-seeking behaviour refers to instances where study participants sought help for personal or emotional problems in the last 12 months from any of the following sources: a phone helpline or the Internet (two items). For example, participants were asked, “Have you sought help for personal or emotional problems from any of these in the last 12 months?” Responses indicating help-seeking from a phone helpline or the Internet were coded as “Yes” and categorised as non-F2F help-seeking. | 1 = Yes, 0 = No | -- |
| Informal help-seeking | Informal help-seeking behaviour refers to instances where study participants sought help for personal or emotional problems in the last 12 months from any of the following sources: a boyfriend/girlfriend/partner, friend, parent, sibling, other relative/family member, another adult, or someone else not listed here (seven items). For example, participants were asked, “Have you sought help for personal or emotional problems from any of these in the last 12 months?” Responses indicating help-seeking from any of these sources were coded as “Yes” and categorised as informal help-seeking. | 1 = Yes, 0 = No | -- |
| Help-seeking from any sources | Any sources refer to instances in which study participants sought help from formal, non-F2F, or informal sources. | 1 = Yes, 0 = No | -- |
| ***Exposure of interest*** |  |  |  |
| Remoteness | The Australian Statistical Geography Standard (ASGS) Remoteness Structure is based on a measure of relative access to services. These remoteness measures are calculated using Accessibility/Remoteness Index of Australia (ARIA+) scores, which are based on the road distance from a populated locality to the nearest urban centre. The lower the ARIA+ score for a populated locality, the greater the access to service. The ASGS Remoteness Structure classifies Australia into five levels of remoteness: i) major cities – the most accessible areas, ii) inner regional – less accessible than major cities, but more accessible than outer regional areas, iii) outer regional – less accessible than inner regional areas, but more accessible than remote areas, iv) remote – less accessible than outer regional areas, but more accessible than very remote areas, and v) very remote – the least accessible areas. In our analyses, we merged the last three categories as “outer regional and remote areas”. | 0 = Major cities,  1 = Inner regional,  2 = Outer regional and remote area | 2 (0.04) |
| ***Factors/predictors*** |  |  |  |
| Child age | In the wave 8 LSAC data, the “baby” cohort (B-cohort) comprises younger adolescents aged 14–15 years, while the “kindergarten” cohort (K-cohort) comprises older adolescents aged 18–19 years. | 1 = 14–15 years  2 = 18–19 years | 0 (0.00) |
| Child sex | The primary caregiver was asked about the “Study children” with the question, “Is the family member male or female?” | 1 = Male,  0 = Female | 0 (0.00) |
| Main language spoken at home | The study children were asked about the main language spoken at home. The question was “Does the family member speak a language other than English at home?” Responses were recoded, with 0 = English as the main language spoken at home, and all other languages categorised as 1 = Non-English. | 1 = Non-English,  0 = English | 140 (2.89) |
| Indigenous status | Study participants were asked about their ethnicity, specifically whether they identified as Aboriginal, Torres Strait Islander, both, or neither. The response options were: 1 = No; 2 = Yes (Aboriginal); 3 = Yes (Torres Strait Islander); and 4 = Yes (both). These responses were then recoded, with '1' converted to '0' to represent “ Non-Indigenous”, and responses '2', '3' and '4' converted to '1', indicating “Indigenous”. This recoding was used to reflect Indigenous status. | 1 = Indigenous, 0 = Non-Indigenous | 0 (0.00%) |
| Primary carer’s financial hardship | Six items were used to assess the financial hardship experienced by the primary caregiver. The primary carer was asked, “In the last 12 months, have any of these happened to you because you were short of money?” The items included i) You could not pay gas, electricity or telephone bills on time, ii) You could not pay the mortgage or rent payments on time, iii) You went without meals, iv) You were unable to heat or cool your home, v) You pawned or sold something because you needed cash, and vi) You sought assistance from a welfare or community organisation. A hardship scale was generated by summing the “Yes” responses to these questions. Responses were then recoded as 0 = “No” and 1-6 = “Yes” to indicate the primary caregiver's financial hardship status. | 1 = Yes, 0 = No | 330 (6.82) |
| Primary carer’s education | Two items were used to assess the primary carer's educational status. First, the primary carer was asked, “What is the level of the highest qualification that you have ever completed?” Responses of “Postgraduate degree” and “Bachelor degree” were coded as 2 = “University degree”. Qualifications such as “Graduate diploma/certificate”, “Advanced diploma/diploma”, “Certificate courses”, and “Other” were coded as 1 = “Diploma/certificate course”. Second, the primary carer was asked, “Have you completed a trade certificate, diploma, degree or any other educational qualification?” Responses of “No” were combined with the previous categories and recoded as 0 = “< 12 years”, representing the primary carer’s educational status. | 0 = < 12 years,  1 = Diploma/certificate,  2 = University degree | 586 (12.11) |
| Socio-Economic Indexes for Areas (SEIFA) | Socio-Economic Indexes for Areas (SEIFA) is a product developed by the Australian Bureau of Statistics (ABS) that ranks areas across Australia according to relative socio-economic advantages and disadvantages. The indixes are based on data collected during the five-yearly Census. In these indexes, we used the relative socioeconomic advantage and disadvantage index. The index variable is rounded to the nearest 10, with deciles scales systematically recoded into five quantiles. The first quantile denotes the most disadvantaged areas, whereas the fifth quantile denotes the least disadvantaged. | 1 = 1^st^ quantile,  2 = 2^nd^ quantile,  3 = 3^rd^ quantile,  4 = 4^th^ quantile,  5 = 5^th^ quantile. | 2 (0.04) |
| General health condition | Study participants were asked to self-report their general health using the question, “In general, how is your health?” This served as a “global health measure” to assess the general health conditions of the participants. LSAC responses were coded as 1 "Excellent", 2 "Very good", 3 "Good", 4 “Fair”, and 5 "Poor". We recoded as 1 = 0 "Excellent", 2 = 1 "Very good", 3 = 2 "Good", 4 or 5 = 3 "Fair or poor", representing the study child’s general health condition. | 0 = Excellent,  1 = Very good,  2 = Good,  3 = Fair or poor | 49 (1.01) |
| Ongoing anxiety or depression | Study participants aged 18–19 years (K-cohort) were asked about their ongoing anxiety disorder or depression. For example, “Do you have any of these ongoing conditions? Anxiety disorder, Depression?” Similarly, the primary carer of the study participants aged 14–15 years (B-cohort) was asked about their children’s ongoing conditions related to anxiety disorder/depression. The question was: “Does the study child have any of these ongoing conditions? ('Ongoing conditions' exist for some period of time (weeks, months or years) or recur regularly. They do not have to be diagnosed by a doctor.) Anxiety disorder, Depression?” In both cases, the responses were coded 0 = “No” and 1 = “Yes”. | 1 = Yes, 0 = No | 40 (0.83) |
| Special health care needs | To assess the special health care needs of the study children, the question was directed to the primary carer for children aged 14–15 years and to the adolescents aged 18–19 years directly. The question was: “Does the child have a condition which has lasted or is expected to last for at least 12 months which causes him/her to use medicine prescribed by a doctor, other than vitamins, or more medical care, mental health or educational services?” Responses were recoded as 1 = “Yes”, and 2 was recoded to 0 = “No”, to reflect the study of children’s special health care needs. | 1 = Yes, 0 = No | 138 (2.85) |
| Suicidal thoughts and behaviours | Four items were used to assess suicidal thoughts and behaviours among participating children: i) having thoughts about suicide, ii) attempting a suicide, iii) making a plan for suicide, and iv) frequency suicide attempts. For instance, participating children were asked “i) have you thought about hurting yourself on purpose, ii) consider attempting suicide, iii) make a plan about how you would attempt suicide, and iv) frequency attempting” to measure their suicidal thoughts and behaviours. Responses to question 'iv' were recategorised, with “zero times” coded as 0 = “No” and 'one or more times' coded as 1= “Yes”. For all items, “Yes” responses were recoded as 1 = “Yes” and “No” responses were recoded as 0 = “No”, to capture the status of suicidal thoughts and behaviours. | 1 = Yes, 0 = No | 53 (1.10) |
| Ever had an alcoholic drink | To assess the status of alcohol consumption among participating children, they were asked, “Have you ever had even part of an alcoholic drink?” The responses for 18–19-year-old adolescents were 1 = “Yes” and 2 = “No”. However, the responses for 14–15 years aged adolescents were 1 = “No”; 2 = “Yes, just a few sips”; 3 = “Yes”, I have had fewer than 10 alcoholic drinks in my life”; 4 = “Yes, I have had 10 or more alcoholic drinks in my life”. Responses for the 14–15-year age group were recoded as 0 = “No” for 1, and 1 = “Yes” for responses 2, 3, and 4. Finally, both age groups were recoded consistently into two categories: 0 = “No” and 1 = “Yes”. | 1 = Yes, 0 = No | 51 (1.05) |
| Ever smoked part of a cigarette | To generate the smoking status of study children, they were asked, “Have you ever smoked even part of a cigarette?” The responses are 1 “Yes” and 2 “No”. We recoded as 1 = 1 “Yes” and 2 = 0 “No”. | 1 = Yes, 0 = No | 26 (0.54) |
| Ever tried any drug | Eight items were assessed to measure whether study participants had ever tried any drug. They were asked, “Have you ever tried for each of the types of drugs listed below: i) cannabis - marijuana/pot/grass/weed/joint, ii) cocaine - coke/charlie/blow/snow, iii) ecstasy - XTC/E/Ex/Eccy/MDMA, iv) hallucinogens - LSD/acid/magic mushrooms, v) sniffing, vi) synthetic cannabis, vii) other psychoactive drug, viii) other illicit drug. For each item, responses were coded “Yes” and “No”. Finally, a single binary indicator was generated: “Yes =1” for any “Yes” response across items; otherwise, “No = 0”. | 1 = Yes, 0 = No | 21 (0.43) |
| Physical activity (Vigorous- intensity) | Study participants were asked, “About how many days each week do you do at least 60 minutes of moderate or vigorous physical activity? This is all the time you spent in activities that increased your heart rate and made you breathe hard,” to assess their physical activity. Responses were categorised according to WHO guidelines to determine whether they met the criteria for physical activity. Those reporting 0 to 4 days of physical activity per week were recoded as 0 = “Does not meet WHO recommendation”, while those reporting 5 or more days were recoded as 1 = “Meet WHO recommendation” [1,2]. | 0 = Does not meet WHO recommendation,  1 = Meet WHO recommendation | 394 (8.15) |
| Screen time (per day) | Screen time, including time spent on TV, computers, and electronic games, was measured for study participants. Aolescents aged 18–19 years were asked, “On average, how much time do you spend playing electronic games per day on weekdays? (Total minutes)” on weekdays. A similar question was asked for weekends. For adolescents aged 14–15 years, their primary carer was asked about the “Total minutes of TV watched on an average week?” and “Total number of electronic gaming minutes for an average week?” The daily screen time (in minutes) for these activities was estimated, and responses were categorised as follows: 0 = None or < 2 hours (0 to 119 minutes) and 1 = ≥ 2 hours (≥ 120 minutes). | 0 = None or < 2 hours,  1 = ≥ 2 hours | 63 (1.30) |
| Social media exposure | Exposure to social media was assessed based on the number of social networks used by study participants. Adolescents were asked, “How many social network accounts do you use once a month or more? (e.g. Facebook, Twitter, Instagram, YouTube.)?” Based on recommendations for Australian children, the number of social networks used by participating children was recoded as: 0 = “None”, 1 = “1 to 4 social networks”, and 2 = “>4 social networks”. | 0 = None  1 = 1 to 4 social networks,  2 = >4 social networks | 51 (1.05) |
| Single parenthood | The primary caregiver was asked about family composition to determine whether they had lived in a single-parent family. For example, parent 1 was asked, “Did you ever live in any of the following family circumstances before you were 18 years of age? In a single-parent family. Another question was also asked: “Parent 1 has a partner?” Then, a single variable, “Single parenthood,” was constructed from the answers to these two questions. Responses were recoded 1 = “Yes” and 0 = “No”. | 1 = Yes, 0 = No | 20 (0.41) |
| Family member with a disability (anyone) | To assess whether any household member had a disability, the following question was asked: “Does someone in the household have a medical condition or disability that has lasted or is expected to last 6 months or restricts their everyday activities?” Responses were coded 0 = “No” and 1 = “Yes”. | 1 = Yes, 0 = No | 29 (0.60) |
| Relationship with parents | To assess the relationship between the participating children and their parents, eight items were used: i) my parents accept me as I am, ii) parents understand me, iii) I trust parents, iv) count on parents, v) parents pay attention, vi) I talk with my parents when I have a problem, vii) parents ask me about problems, and viii) share feelings with parents. For each statement, the participating children rated their relationship with their parents on a 4-point scale: 1 = “Almost never or never true”, 2 = “Sometimes true”, 3 = “Often true”, and 4 = “Almost always or always true”. The mean score across the eight items was derived, and relationships were categorised into two groups: bottom 25% were classified as “poor”, and the top 75% as “good”. | 0 = Poor (bottom 25%)  1 = Good (top 75%) | 47 (0.97) |
| Bullying victimisation | Study participants reported their experiences of bullying victimisation across eight itmes in three settings: workplace, study place, and other locations. The items were: i) inappropriate jokes or teasing, ii) freezing out, ignoring or excluding, iii) repeated criticism and humiliation, iv) unreasonable pressure to produce work, v) threats to person or property, verbally or physically, vi) withholding information which affects work/study performance, vii) attempts to belittle work and efforts to contribute, and viii) spreading gossip and rumours. For example, the study participants were asked “Where did your experience of making inappropriate jokes or teasing happen? Workplace”. Bullying victimisation was coded as “Yes” if participants reported experiencing any type of bullying in any of the three settings. | 1 = Yes, 0 = No | 76 (1.57) |
| Discrimination/ unfair treatment | Eight items were used to assess discrimination or unfair treatment based on the following factors: language or accent, colour of skin, disability, religious beliefs, cultural background, mental health problems, body size/appearance, sex, and sex identity. For example, the participating children were asked, “In the last 6 months have you been treated unfairly or badly because of your language or accent?”. If they reported “Yes” to any of these items, they were categorised as having experienced discrimination or unfair treatment due to one or more of the specified reasons. Otherwise, they were categorised as “No”. | 1 = Yes, 0 = No | 45 (0.93) |
| Participation in community groups | Participating children were asked about unpaid voluntary work they had performed in the last 12 months, covering 13 items: coaching, school/education, community/welfare, church or religious groups, emergency services, youth/mentoring, cultural, health, teaching/training, animal welfare, environment, immigrant/refugee assistance, international aid/development, human rights, professional associations, ethnic societies, and other. For example, they were asked, “In the last 12 months, did you do any unpaid voluntary work for any of these types of organisations? Sport/recreation (e.g. coaching, refereeing)”. If participants answered “Yes” to any of the items, they were coded as “Yes”, indicating participation in community groups. Otherwise, they were coded as “No”. | 1 = Yes, 0 = No | 37 (0.76) |
| Active in religious or spiritual groups | Study participants were asked, “Are you active in a religious or spiritual group, such as regularly going to services, Sunday school or a religious youth club?/ or activities or meetings?” Responses of “Yes” were coded as indicating active participation in religious or spiritual groups, while other responses were coded as “No”. | 1 = Yes, 0 = No | 40 (0.83) |

**Table S2** Justification of selected predictors associated with mental health help-seeking behaviours based on previous literature

| **Variables/Indicators** | **Variables name** | **References** |
| --- | --- | --- |
| **Outcome variables** | Formal help-seeking | [3-5] |
|  | Non-F2F help-seeking | [4] |
|  | Informal help-seeking | [4,5] |
|  | Help-seeking from any sources | Any of the above three |
| **Exposure of interest** | Remoteness | [6-9] |
| **Factors/Predictors** |  |  |
| Demographics | Child age | [4,10] |
|  | Child sex | [3,4, 10-13] |
|  | Main language spoken at home | [3,10,14] |
|  | Indigenous status | [3,6,14,15] |
| Socio-economic factors | Primary carer’s financial hardship | [16,17] |
|  | Primary carer’s education | [15,18-21] |
|  | Socio-Economic Indexes for Areas (SEIFA) | [6,17] |
| Health and well-being | General health condition | [21] |
|  | Ongoing anxiety or depression | [4] |
|  | Special health care needs | [22] |
|  | Suicidal thoughts and behaviours | [9,23] |
| Lifestyle-related factors | Ever had an alcoholic drink | [22,24] |
|  | Ever smoked part of a cigarette | [25] |
|  | Ever tried any drug | [22,24] |
|  | Physical activity (Vigorous- intensity) | [1,26] |
|  | Screen time (per day) | [27,28] |
|  | Social media exposure | [27,29] |
| Parent/family related factors | Single parenthood | [3] |
|  | Family member with a disability (anyone) | [22,30] |
|  | Relationship with parents | [3,4] |
| Social/community-related factors | Bullying victimisation | [3,31-33] |
|  | Discrimination/unfair treatment | [3] |
|  | Participation in community groups | [34-36] |
|  | Active in religious or spiritual groups | [30] |

**Table S3** Prevalence of help-seeking behaviour among adolescents for specific indicators of the four broader sources, by age, sex, and remoteness (weighted)

| **Help-seeking sources** | **Prevalence of help-seeking behaviour; % (95% CI)** | | | |
| --- | --- | --- | --- | --- |
|  | **Major cities**  **(N = 3,314)** | **Inner regional**  **(N = 1,000)** | **Outer regional or remote (N = 521)** | **Overall (N = 4,837) ^a^** |
| **Formal** | 34.89 (33.05–36.77) | 35.16 (31.8–38.68) | 29.8 (25.46–34.54) | 34.42 (32.9–35.98) |
| By age |  |  |  |  |
| - 14–15 years | 37.51 (34.82–40.27) | 37.75 (33.29–42.42) | 32.11 (26.31–38.52) | 36.96 (34.79–39.18) |
| - 18–19 years | 32.2 (29.74–34.76) | 31.6 (26.61–37.05) | 26.73 (20.49–34.05) | 31.6 (29.49–33.79) |
| By sex |  |  |  |  |
| - Female | 37.89 (35.28–40.57) | 38.3 (33.76–43.07) | 37.7 (31.15–44.72) | 37.97 (35.82–40.18) |
| - Male* | 31.76 (29.21–34.42) | 31.18 (26.29–36.51) | 23.06 (17.6–29.61) | 30.65 (28.53–32.86) |
| *Teacher* | 20.31 (18.78–21.93) | 20.63 (17.93–23.61) | 16.78 (13.44–20.75) | 20.01 (18.75–21.33) |
| By age |  |  |  |  |
| - 14–15 years | 24.96 (22.60–27.48) | 25.61 (21.78–29.86) | 19.29 (14.91–24.58) | 24.47 (22.58–26.46) |
| - 18–19 years | 15.55 (13.72–17.57) | 13.76 (10.45–17.91) | 13.44 (8.71–20.18) | 15.03 (13.46–16.74) |
| By sex |  |  |  |  |
| - Female | 20.06 (17.94–22.36) | 19.54 (16.16–23.43) | 21.02 (15.82–27.39) | 20.03 (18.29–21.89) |
| - Male | 20.57 (18.42–22.90) | 22.00 (17.83–26.83) | 13.15 (9.21–18.45) | 19.98 (18.19–21.90) |
|  |  |  |  |  |
| *Other school staff* | 10.13 (8.98–11.41) | 9.13 (7.23–11.47) | 11.00 (8.11–14.74) | 10.02 (9.06–11.07) |
| By age |  |  |  |  |
| - 14–15 years | 14.55 (12.63–16.70) | 13.49 (10.48–17.19) | 14.65 (10.46–20.13) | 14.33 (12.77–16.04) |
| - 18–19 years | 5.61 (4.49–6.97) | 3.14 (1.80–5.44) | 6.13 (3.06–11.90) | 5.22 (4.28–6.34) |
| By sex |  |  |  |  |
| - Female | 10.69 (9.07–12.57) | 11.36 (8.60–14.87) | 12.66 (8.51–18.44) | 11.02 (9.65–12.55) |
| - Male | 9.55 (8.00–11.36) | 6.31 (4.11–9.57) | 9.58 (5.96–15.03) | 8.96 (7.68–10.43) |
|  |  |  |  |  |
| *Family doctor/GP* | 9.46 (8.39–10.64) | 9.35 (7.44–11.69) | 7.65 (5.28–10.97) | 9.26 (8.37–10.24) |
| By age |  |  |  |  |
| - 14–15 years | 6.03 (4.76–7.62) | 6.62 (4.62–9.39) | 4.24 (2.30–7.71) | 5.96 (4.93–7.18) |
| - 18–19 years | 12.97 (11.33–14.81) | 13.11 (9.75–17.41) | 12.18 (7.69–18.77) | 12.95 (11.50–14.55) |
| By sex |  |  |  |  |
| - Female | 12.22 (10.60–14.05) | 11.02 (8.42–14.31) | 10.76 (6.97–16.25) | 11.85 (10.51–13.34) |
| - Male | 6.57 (5.30–8.13) | 7.23 (4.71–10.94) | 5.00 (2.49–9.77) | 6.51 (5.41–7.82) |
|  |  |  |  |  |
| *Mental health professional** | 12.20 (10.97–13.54) | 11.40 (9.27–13.96) | 7.72 (5.39–10.93) | 11.58 (10.57–12.66) |
| By age |  |  |  |  |
| - 14–15 years | 10.93 (9.25–12.87) | 8.62 (6.35–11.60) | 6.35 (3.86–10.29) | 9.91 (8.61–11.38) |
| - 18–19 years | 13.50 (11.78–15.43) | 15.24 (11.50–19.91) | 9.54 (5.69–15.54) | 13.43 (11.92–15.10) |
| By sex |  |  |  |  |
| - Female | 14.93 (13.10–16.97) | 15.08 (11.87–18.98) | 13.53 (9.08–19.70) | 14.83 (13.29–16.51) |
| - Male* | 9.35 (7.82–11.15) | 6.74 (4.48–10.03) | 2.76 (1.33–5.63) | 8.12 (6.91–9.51) |
| **Non-F2F*** | 19.69 (18.24–21.23) | 16.02 (13.56–18.82) | 16.66 (13.2–20.81) | 18.64 (17.44–19.91) |
| By age |  |  |  |  |
| - 14–15 years | 16.49 (14.57–18.61) | 13.41 (10.57–16.88) | 12.62 (9.02–17.38) | 15.38 (13.86–17.04) |
| - 18–19 years* | 22.97 (20.83–25.26) | 19.6 (15.52–24.44) | 22.04 (16.02–29.53) | 22.28 (20.43–24.24) |
| By sex |  |  |  |  |
| - Female* | 22.01 (19.92–24.25) | 17.00 (13.62–21.02) | 23.96 (18.12–30.98) | 21.10 (19.35–22.95) |
| - Male* | 17.27 (15.31–19.44) | 14.77 (11.46–18.83) | 10.43 (7–15.27) | 16.03 (14.43–17.77) |
| *Phone help line* | 2.94 (2.37–3.64) | 2.15 (1.30–3.54) | 4.09 (2.21–7.43) | 2.90 (2.39–3.51) |
| By age |  |  |  |  |
| - 14–15 years | 3.42 (2.55–4.56) | 1.75 (0.92–3.30) | 2.91 (1.27–6.52) | 2.99 (2.32–3.85) |
| - 18–19 years | 2.45 (1.77–3.36) | 2.71 (1.26–5.71) | 5.66 (2.38–12.87) | 2.79 (2.08–3.74) |
| By sex |  |  |  |  |
| - Female | 3.61 (2.79–4.65) | 2.85 (1.63–4.94) | 6.48 (3.06–13.20) | 3.71 (2.94–4.66) |
| - Male | 2.24 (1.52–3.29) | 1.26 (0.41–3.80) | 2.05 (0.75–5.48) | 2.04 (1.44–2.87) |
| *Internet** | 18.05 (16.66–19.54) | 14.66 (12.31–17.37) | 16.29 (12.86–20.42) | 17.19 (16.03–18.41) |
| By age |  |  |  |  |
| - 14–15 years | 14.49 (12.69–16.49) | 12.23 (9.51–15.59) | 11.97 (8.47–16.65) | 13.71 (12.27–15.28) |
| - 18-19 years* | 21.71 (19.63–23.95) | 18.00 (14.1–22.7) | 22.04 (16.02–29.53) | 21.07 (19.27–22.99) |
| By sex |  |  |  |  |
| - Female* | 20.35 (18.32–22.53) | 14.84 (11.68–18.68) | 23.15 (17.37–30.15) | 19.40 (17.72–21.21) |
| - Male | 15.66 (13.80–17.73) | 14.43 (11.15–18.47) | 10.43 (7.00–15.27) | 14.84 (13.31–16.51) |
| **Informal** | 95.95 (95.07–96.68) | 96.20 (94.63–97.33) | 94.42 (91.46–96.4) | 95.84 (95.12–96.46) |
| By age |  |  |  |  |
| - 14–15 years | 95.55 (94.29–96.54) | 96.56 (94.76–97.75) | 94.42 (89.96–96.97) | 95.64 (94.66–96.45) |
| - 18–19 years | 96.37 (95.01–97.36) | 95.72 (92.51–97.59) | 94.42 (89.8–97.02) | 96.07 (94.94–96.96) |
| By sex |  |  |  |  |
| - Female | 97.16 (96.05–97.96) | 97.74 (96.03–98.73) | 98.38 (96.35–99.29) | 97.4 (96.57–98.03) |
| - Male | 94.69 (93.22–95.86) | 94.25 (91.16–96.31) | 91.05 (85.78–94.49) | 94.19 (92.95–95.23) |
| *Boyfriend/girlfriend/partner** | 32.61 (30.79–34.47) | 39.73 (36.24–43.34) | 38.11 (33.32–43.15) | 34.61 (33.07–36.19) |
| By age |  |  |  |  |
| - 14–15 years* | 19.71 (17.52–22.10) | 28.60 (24.37–33.25) | 28.94 (23.11–35.56) | 22.69 (20.79–24.71) |
| - 18–19 years* | 45.83 (43.12–48.56) | 55.05 (49.53–60.46) | 50.32 (42.62–58.02) | 47.91 (45.58–50.25) |
| By sex |  |  |  |  |
| - Female* | 33.74 (31.19–36.39) | 42.58 (37.86–47.44) | 37.96 (31.32–45.09) | 36.07 (33.92–38.28) |
| - Male | 31.42 (28.89–34.07) | 36.12 (31.03–41.55) | 38.24 (31.52–45.43) | 33.06 (30.88–35.31) |
| *Friend** | 81.19 (79.56–82.71) | 77.97 (74.88–80.77) | 75.93 (71.12–80.16) | 80.00 (78.64–81.30) |
| By age |  |  |  |  |
| - 14–15 years | 76.85 (74.41–79.12) | 78.02 (74.01–81.57) | 72.65 (66.14–78.32) | 76.64 (74.68–78.49) |
| - 18–19 years* | 85.63 (83.41–87.60) | 77.89 (72.86–82.22) | 80.29 (72.67–86.19) | 83.76 (81.83–85.53) |
| By sex |  |  |  |  |
| - Female* | 86.38 (84.26–88.25) | 82.84 (78.95–86.14) | 86.02 (80.16–90.36) | 85.58 (83.86–87.15) |
| - Male* | 75.77 (73.25–78.12) | 71.79 (66.74–76.35) | 67.31 (60.06–73.83) | 74.07 (71.93–76.11) |
| *Parent* | 74.15 (72.36–75.86) | 74.46 (71.14–77.52) | 69.95 (64.98–74.49) | 73.78 (72.29–75.22) |
| By age |  |  |  |  |
| - 14–15 years | 75.96 (73.49–78.26) | 76.28 (72.06–80.04) | 67.77 (60.98–73.89) | 75.11 (73.10–77.03) |
| - 18–19 years | 72.30 (69.66–74.78) | 71.95 (66.46–76.85) | 72.84 (65.34–79.23) | 72.30 (70.06–74.43) |
| By sex |  |  |  |  |
| - Female | 75.54 (73.02–77.90) | 75.72 (71.20–79.73) | 75.98 (69.67–81.33) | 75.63 (73.58–77.57) |
| - Male | 72.70 (70.10–75.15) | 72.86 (67.77–77.41) | 64.80 (57.40–71.55) | 71.82 (69.62–73.92) |
| *Sibling* | 42.15 (40.23–44.09) | 40.55 (37.10–44.09) | 42.98 (38.06–48.05) | 41.92 (40.33–43.53) |
| By age |  |  |  |  |
| - 14–15 years | 37.47 (34.79–40.24) | 34.01 (29.73–38.57) | 36.84 (30.63–43.52) | 36.64 (34.48–38.86) |
| - 18–19 years | 46.94 (44.22–49.67) | 49.55 (44.04–55.06) | 51.16 (43.43–58.83) | 47.81 (45.48–50.14) |
| By sex |  |  |  |  |
| - Female | 47.39 (44.67–50.12) | 44.42 (39.74–49.21) | 48.56 (41.47–55.71) | 46.86 (44.62–49.12) |
| - Male | 36.68 (34.04–39.40) | 35.64 (30.65–40.96) | 38.22 (31.62–45.29) | 36.66 (34.45–38.94) |
| *Other relatives/family members* | 26.92 (25.21–28.71) | 30.48 (27.23–33.92) | 28.09 (23.73–32.91) | 27.77 (26.33–29.26) |
| By age |  |  |  |  |
| - 14–15 years | 27.17 (24.71–29.78) | 30.71 (26.48–35.28) | 23.76 (18.54–29.90) | 27.56 (25.56–29.66) |
| - 18–19 years | 26.68 (24.35–29.14) | 30.16 (25.25–35.57) | 33.86 (26.83–41.69) | 28.00 (25.95–30.15) |
| By sex |  |  |  |  |
| - Female* | 27.31 (24.94–29.81) | 34.25 (29.79–39.00) | 33.03 (26.68–40.05) | 29.37 (27.35–31.47) |
| - Male | 26.52 (24.10–29.10) | 25.70 (21.18–30.80) | 23.88 (18.15–30.75) | 26.07 (24.03–28.22) |
| *Other adults** | 11.55 (10.33–12.89) | 14.26 (11.93–16.96) | 14.16 (10.99–18.07) | 12.36 (11.32–13.49) |
| By age |  |  |  |  |
| - 14–15 years* | 10.36 (8.71–12.27) | 14.94 (11.86–18.65) | 11.49 (7.98–16.28) | 11.49 (10.10–13.05) |
| - 18–19 years | 12.78 (11.05–14.74) | 13.33 (10.02–17.51) | 17.72 (12.42–24.64) | 13.33 (11.81–15.02) |
| By sex |  |  |  |  |
| - Female* | 12.24 (10.47–14.25) | 16.02 (12.68–20.04) | 19.60 (14.20–26.42) | 13.73 (12.18–15.44) |
| - Male | 10.84 (9.24–12.68) | 12.04 (9.16–15.67) | 9.52 (6.39–13.95) | 10.90 (9.57–12.40) |
| *Someone else not listed above* | 1.97 (1.48–2.60) | 1.88 (1.17–3.01) | 3.24 (1.85–5.59) | 2.08 (1.66–2.60) |
| By age |  |  |  |  |
| - 14–15 years | 2.41 (1.66–3.48) | 2.03 (1.06–3.84) | 4.18 (2.12–8.08) | 2.52 (1.88–3.37) |
| - 18–19 years | 1.51 (0.99–2.31) | 1.68 (0.87–3.25) | 1.97 (0.77–4.99) | 1.59 (1.13–2.22) |
| By sex |  |  |  |  |
| - Female | 2.27 (1.58–3.26) | 1.24 (0.63–2.43) | 2.39 (1.06–5.28) | 2.06 (1.52–2.78) |
| - Male | 1.65 (1.06–2.56) | 2.70 (1.43–5.05) | 3.96 (1.89–8.10) | 2.10 (1.52–2.91) |
| **Any sources** | 97.26 (96.52–97.84) | 98.03 (96.73–98.82) | 96.07 (93.52–97.64) | 97.29 (96.7–97.78) |
| By age |  |  |  |  |
| - 14–15 years | 97.18 (96.15–97.93) | 98.44 (96.96–99.21) | 96.58 (93.03–98.35) | 97.39 (96.6–97.99) |
| - 18–19 years | 97.34 (96.18–98.16) | 97.46 (94.7–98.8) | 95.38 (90.75–97.75) | 97.18 (96.21–97.91) |
| By sex |  |  |  |  |
| - Female | 97.94 (97.02–98.58) | 98.48 (96.86–99.27) | 98.63 (96.67–99.45) | 98.12 (97.43–98.63) |
| - Male | 96.55 (95.30–97.47) | 97.46 (94.91–98.75) | 93.87 (89.28–96.57) | 96.41 (95.37–97.22) |

CI = Confidence interval, Non-F2F = Non-face-to-face, ^a^ Two (2) missing cases for remoteness, *Statistically significant at 5% level of significance

**Table S4** Prevalence of adolescents’ help-seeking behaviours from informal and non-F2F sources, by predictors

| **Factors/Predictors** |  | **Informal** | | **Non-F2F** | |
| --- | --- | --- | --- | --- | --- |
|  | **N** | **% (n)** | **p-value** | **% (n)** | **p-value** |
| ***Demographics*** |  |  |  |  |  |
| Age |  |  |  |  |  |
| - 14–15 years | 2541 | 95.55 (2,428) | 0.051 | 16.10 (409) | <0.001 |
| - 18–19 years | 2296 | 96.65 (2,219) |  | 23.13 (531) |  |
| Sex |  |  |  |  |  |
| - Female | 2513 | 97.37 (2,447) | <0.001 | 21.73 (546) | <0.001 |
| - Male | 2324 | 94.66 (2,200) |  | 16.95 (394) |  |
| Main language spoken at home |  |  |  |  |  |
| - English | 4388 | 96.19 (4,221) | 0.722 | 19.19 (842) | 0.057 |
| - Non-English | 309 | 95.79 (296) |  | 23.62 (73) |  |
| - Missing | 140 | 92.86 (130) |  | 17.86 (25) |  |
| Indigenous status |  |  |  |  |  |
| - Non-Indigenous | 4729 | 96.09 (4,544) | 0.704 | 19.50 (922) | 0.462 |
| - Indigenous | 108 | 95.37 (103) |  | 16.67 (18) |  |
| ***Socio-economic status*** |  |  |  |  |  |
| Primary carer’s financial hardship |  |  |  |  |  |
| - No | 3842 | 96.23 (3,697) | 0.275 | 19.83 (762) | 0.047 |
| - Yes | 665 | 95.34 (634) |  | 16.54 (110) |  |
| - Missing | 330 | 95.76 (316) |  | 20.61 (68) |  |
| Primary carer’s education |  |  |  |  |  |
| - <12 years | 597 | 95.31 (569) | 0.670 | 17.76 (106) | <0.001 |
| - Diploma/certificate | 2299 | 96.00 (2,207) |  | 17.40 (400) |  |
| - University degree | 1355 | 96.16 (1,303) |  | 23.10 (313) |  |
| - Missing | 586 | 96.93 (568) |  | 20.65 (121) |  |
| SEIFA |  |  |  |  |  |
| - First quantile (q1, most disadvantaged) | 632 | 95.25 (602) | 0.528 | 15.98 (101) | 0.001 |
| - Second quantile (q2) | 864 | 95.60 (826) |  | 17.82 (154) |  |
| - Third quantile (q3) | 934 | 96.25 (899) |  | 17.88 (167) |  |
| - Fourth quantile (q4) | 1117 | 96.78 (1,081) |  | 19.52 (218) |  |
| - Fifth quantile (q5, least disadvantaged) | 1288 | 96.04 (1,237) |  | 23.29 (300) |  |
| - Missing | 2 | 100.00 (2) |  | 0.00 (0) |  |
| Remoteness |  |  |  |  |  |
| - Major cities | 3314 | 96.23 (3,189) | 0.412 | 21.00 (696) | <0.001 |
| - Inner regional | 1000 | 96.10 (961) |  | 16.00 (160) |  |
| - Outer regional and remote | 521 | 95.01 (495) |  | 16.12 (84) |  |
| - Missing | 2 | 100.00 (2) |  | 0.00 (0) |  |
| ***Health and well-being*** |  |  |  |  |  |
| General health condition |  |  |  |  |  |
| - Excellent | 893 | 96.42 (861) | <0.001 | 16.57 (148) | <0.001 |
| - Very good | 2017 | 96.98 (1,956) |  | 16.96 (342) |  |
| - Good | 1415 | 95.90 (1,357) |  | 22.19 (314) |  |
| - Fair or poor | 463 | 91.58 (424) |  | 26.57 (123) |  |
| - Missing | 49 | 100.00 (49) |  | 26.53 (13) |  |
| Ongoing anxiety or depression |  |  |  |  |  |
| - No | 4061 | 95.89 (3,894) | 0.150 | 17.53 (712) | <0.001 |
| - Yes | 736 | 97.01 (714) |  | 30.03 (221) |  |
| - Missing | 40 | 97.50 (39) |  | 17.50 (7) |  |
| Special health care needs |  |  |  |  |  |
| - No | 3845 | 96.02 (3,692) | 0.752 | 18.78 (722) | 0.064 |
| - Yes | 854 | 96.25 (822) |  | 21.55 (184) |  |
| - Missing | 138 | 96.38 (133) |  | 24.64 (34) |  |
| Suicidal thoughts and behaviours |  |  |  |  |  |
| - No | 3712 | 96.34 (3,576) |  | 15.27 (567) | <0.001 |
| - Yes | 1072 | 95.06 (1019) |  | 33.96 (364) |  |
| - Missing | 53 | 98.11 (52) | 0.058 | 16.98 (9) |  |
| **Lifestyle-related factors** |  |  |  |  |  |
| Ever had an alcoholic drink |  |  |  |  |  |
| - No | 1328 | 95.03 (1,262) | 0.025 | 13.48 (179) | <0.001 |
| - Yes | 3458 | 96.44 (3,335) |  | 21.78 (753) |  |
| - Missing | 51 | 98.04 (50) |  | 15.69 (8) |  |
| Ever smoked part of a cigarette |  |  |  |  |  |
| - No | 3493 | 95.76 (3,345) | 0.095 | 17.66 (617) | <0.001 |
| - Yes | 1318 | 96.81 (1,276) |  | 24.13 (318) |  |
| - Missing | 26 | 100.00 (26) |  | 19.23 (5) |  |
| Ever tried any drug |  |  |  |  |  |
| - No | 3615 | 95.60 (3,456) | 0.005 | 17.54 (634) | <0.001 |
| - Yes | 1201 | 97.42 (1,170) |  | 25.40 (305) |  |
| - Missing | 21 | 100.00 (21) |  | 4.76 (1) |  |
| Physical activity (Vigorous- intensity) |  |  |  |  |  |
| - Does not meet WHO recommendations | 3369 | 96.29 (3,244) | 0.764 | 19.47 (656) | 0.008 |
| - Meet WHO recommendations | 1074 | 96.09 (1,032) |  | 15.83 (170) |  |
| - Missing | 394 | 94.16 (371) |  | 28.93 (114) |  |
| Screen time (per day) |  |  |  |  |  |
| - None or <2 hours | 2860 | 96.68 (2,765) | 0.010 | 18.95 (542) | 0.298 |
| - ≥2 hours | 1914 | 95.19 (1,822) |  | 20.17 (386) |  |
| - Missing | 63 | 95.24 (60) |  | 19.05 (12) |  |
| Social media exposure |  |  |  |  |  |
| - None | 109 | 94.50 (103) | 0.344 | 10.09 (11) | <0.001 |
| - 1 to 4 social networks | 3585 | 95.90 (3,438) |  | 16.74 (600) |  |
| - >4 social networks | 1092 | 96.70 (1,056) |  | 29.30 (320) |  |
| - Missing | 51 | 98.04 (50) |  | 17.65 (9) |  |
| ***Parent/family-related factors*** |  |  |  |  |  |
| Single parenthood |  |  |  |  |  |
| - No | 3850 | 96.26 (3,706) | 0.146 | 18.88 (727) | 0.055 |
| - Yes | 967 | 95.24 (921) |  | 21.61 (209) |  |
| - Missing | 20 | 100.00 (20) |  | 20.00 (4) |  |
| Family member with a disability (anyone) |  |  |  |  |  |
| - No | 2886 | 96.47 (2,784) | 0.069 | 19.61 (566) | 0.723 |
| - Yes | 1922 | 95.42 (1,834) |  | 19.20 (369) |  |
| - Missing | 29 | 100.00 (29) |  | 17.24 (5) |  |
| Relationship with parents |  |  |  |  |  |
| - Poor (bottom 25%) | 1348 | 91.99 (1,240) | <0.001 | 27.97 (377) | <0.001 |
| - Good (top 75%) | 3442 | 97.62 (3,360) |  | 16.12 (555) |  |
| - Missing | 47 | 100.00 (47) |  | 17.02 (8) |  |
| ***Social/community-related factors*** |  |  |  |  |  |
| Bullying victimisation |  |  |  |  |  |
| - No | 2858 | 95.56 (2,731) | 0.032 | 16.86 (482) | <0.001 |
| - Yes | 1903 | 96.79 (1,842) |  | 23.49 (447) |  |
| - Missing | 76 | 97.37 (74) |  | 14.47 (11) |  |
| Discrimination/unfair treatment |  |  |  |  |  |
| - No | 3343 | 96.17 (3,215) | 0.534 | 16.27 (544) | <0.001 |
| - Yes | 1449 | 95.79 (1,388) |  | 26.78 (388) |  |
| - Missing | 45 | 97.78 (44) |  | 17.78 (8) |  |
| Participation in community groups |  |  |  |  |  |
| - No | 2413 | 95.11 (2,295) | 0.001 | 18.61 (449) | 0.154 |
| - Yes | 2387 | 96.98 (2,315) |  | 20.23 (483) |  |
| - Missing | 37 | 100.00 (37) |  | 21.62 (8) |  |
| Active in a religious or spiritual group |  |  |  |  |  |
| - No | 4074 | 95.90 (3,907) | 0.234 | 19.83 (808) | 0.077 |
| - Yes | 723 | 96.82 (700) |  | 17.01 (123) |  |
| - Missing | 40 | 100.00 (40) |  | 22.50 (9) |  |

Non-F2F = Non-face-to-face, SEIFA = Socio-Economic Index for Areas, WHO = World Health Organisation

**Table S5** Factor associated with non-F2F and informal help-seeking behaviours among adolescents in Australia (N=4837)

| **Factors/Predictors** | **OR (95% CI)** | | | |
| --- | --- | --- | --- | --- |
|  | **Non-F2F** | | **Informal** | |
|  | **Unadjusted (4,837)** | **Adjusted** | **Unadjusted** | **Adjusted** |
| Age |  |  |  |  |
| - 18–19 years | 1.57 (1.36–1.81)* | 1.33 (1.06–1.66)* | 1.34 (1.00–1.80) | 1.25 (0.83–1.88) |
| - Ref. 14–15 years |  |  |  |  |
| Sex |  |  |  |  |
| - Male | 0.74 (0.64–0.85)* | 0.87 (0.73–1.02) | 0.48 (0.35–0.65)* | 0.53 (0.37–0.76)* |
| - Ref. Female |  |  |  |  |
| Main language spoken at home |  |  |  |  |
| - Non-English | 1.30 (0.99–1.71) | 1.25 (0.94–1.66) | 0.90 (0.51–1.60) | 1.03 (0.55–1.92) |
| - Ref. English |  |  |  |  |
| Indigenous status |  |  |  |  |
| - Indigenous | 0.83 (0.50–1.38) | 0.72 (0.37–1.40) | 0.84 (0.34–2.08) | 0.91 (0.31–2.70) |
| - Ref. Non-Indigenous |  |  |  |  |
| Primary carer’s financial hardship |  |  |  |  |
| - Yes | 0.83 (0.67–1.03) | 0.79 (0.62–1.01) | 0.83 (0.56–1.24) | 1.01 (0.63–1.62) |
| - Ref. No |  |  |  |  |
| Primary carer’s education |  |  |  |  |
| - Diploma/certificate | 0.97 (0.77–1.23) | 1.00 (0.76–1.32) | 1.17 (0.76–1.79) | 1.17 (0.72–1.92) |
| - University degree | 1.33 (1.04–1.69)* | 1.24 (0.94–1.63) | 1.22 (0.77–1.93) | 1.07 (0.61–1.87) |
| - Ref. <12 years |  |  |  |  |
| SEIFA |  |  |  |  |
| - Second quantile (q2) | 1.14 (0.87–1.50) | 1.03 (0.76–1.41) | 1.08 (0.66–1.77) | 1.09 (0.62–1.91) |
| - Third quantile (q3) | 1.14 (0.87–1.50) | 1.26 (0.94–1.70) | 1.28 (0.78–2.11) | 1.23 (0.71–2.12) |
| - Fourth quantile (q4) | 1.27 (0.98–1.65) | 1.18 (0.87–1.61) | 1.50 (0.91–2.45) | 1.37 (0.75–2.48) |
| - Fifth quantile (q5, least disadvantaged) | 1.60 (1.24–2.05)* | 1.53 (1.13–2.08)* | 1.21 (0.76–1.92) | 0.87 (0.49–1.56) |
| - Ref. First quantile (q1, most disadvantaged) |  |  |  |  |
| Remoteness |  |  |  |  |
| - Inner Regional | 0.72 (0.59–0.87)* | 0.84 (0.65–1.08) | 0.97 (0.67–1.39) | 1.02 (0.65–1.60) |
| - Outer Regional and Remote | 0.72 (0.56–0.93)* | 0.85 (0.62–1.16) | 0.75 (0.48–1.15) | 0.74 (0.41–1.34) |
| - Ref. Major cities |  |  |  |  |
| General health condition |  |  |  |  |
| - Very good | 1.03 (0.83–1.27) | 0.85 (0.66–1.08) | 1.19 (0.77–1.85) | 1.24 (0.75–2.04) |
| - Good | 1.43 (1.16–1.78)* | 0.92 (0.72–1.18) | 0.87 (0.56–1.36) | 1.08 (0.63–1.85) |
| - Fair or poor | 1.81 (1.38–2.37)* | 0.79 (0.56–1.11) | 0.41 (0.25–0.66)* | 0.53 (0.28–1.00) |
| - Ref. Excellent |  |  |  |  |
| Ongoing anxiety or depression (Ref. No) |  |  |  |  |
| - Yes | 2.01 (1.68–2.40)* | 1.31 (1.03–1.65)* | 1.40 (0.89–2.20) | 1.92 (1.07–3.44)* |
| - Ref. No |  |  |  |  |
| Special health care needs |  |  |  |  |
| - Yes | 1.21 (1.00–1.45)* | 0.92 (0.72–1.16) | 1.06 (0.72–1.56) | 1.02 (0.64–1.62) |
| - Ref. No |  |  |  |  |
| Suicidal thoughts and behaviours |  |  |  |  |
| - Yes | 2.84 (2.43–3.31)* | 2.05 (1.66–2.53)* | 0.73 (0.53–1.01) | 0.9 (0.62–1.31) |
| - Ref. No |  |  |  |  |
| Ever had alcoholic drink |  |  |  |  |
| - Yes | 1.78 (1.49–2.12)* | 1.27 (0.99–1.63) | 1.40 (1.03–1.91)* | 1.15 (0.75–1.75) |
| - Ref. No |  |  |  |  |
| Ever had smoked part of a cigarette |  |  |  |  |
| - Yes | 1.48 (1.27–1.73)* | 0.92 (0.73–1.16) | 1.35 (0.95–1.91) | 0.92 (0.55–1.53) |
| - Ref. No |  |  |  |  |
| Ever tried any drug |  |  |  |  |
| - Yes | 1.60 (1.37–1.87)* | 1.19 (0.95–1.49) | 1.74 (1.18–2.57)* | 1.88 (1.05–3.37)* |
| - Ref. No |  |  |  |  |
| Physical activity (Vigorous- intensity) |  |  |  |  |
| - Meet WHO recommendations | 0.78 (0.65–0.94)* | 0.87 (0.71–1.07) | 0.95 (0.66–1.35) | 1.01 (0.66–1.55) |
| - Ref. Does not meet WHO recommendations) |  |  |  |  |
| Screen time (per day) |  |  |  |  |
| - ≥2 hours | 1.07 (0.93–1.24) | 1.06 (0.88–1.26) | 0.67 (0.50–0.90)* | 0.95 (0.66–1.35) |
| - Ref. None or <2 hours |  |  |  |  |
| Social media exposure |  |  |  |  |
| - 1 to 4 social networks | 1.79 (0.96–3.37) | 1.41 (0.71–2.80) | 1.36 (0.59–3.15) | 1.38 (0.50–3.78) |
| - >4 social networks | 3.67 (1.94–6.93)* | 2.45 (1.20–5.03)* | 1.71 (0.70–4.16) | 2.05 (0.69–6.11) |
| - Ref. None |  |  |  |  |
| Single parenthood |  |  |  |  |
| - Yes | 1.18 (0.99–1.41) | 1.12 (0.93–1.35) | 0.78 (0.55–1.09) | 0.75 (0.50–1.12) |
| - Ref. No |  |  |  |  |
| Family member with a disability (anyone) |  |  |  |  |
| - Yes | 0.97 (0.84–1.13) | 0.93 (0.79–1.10) | 0.76 (0.57–1.02) | 0.77 (0.56–1.05) |
| - Ref. No |  |  |  |  |
| Relationship with parents |  |  |  |  |
| - Good (top 75%) | 0.50 (0.43–0.58)* | 0.72 (0.61–0.85)* | 3.56 (2.65–4.78)* | 3.91 (2.73–5.60)* |
| - Ref. Poor (bottom 25%) |  |  |  |  |
| Bullying victimisation |  |  |  |  |
| - Yes | 1.51 (1.31–1.75)* | 1.12 (0.94–1.34) | 1.41 (1.03–1.92)* | 1.76 (1.16–2.65)* |
| - Ref. No |  |  |  |  |
| Discrimination/unfair treatment |  |  |  |  |
| - Yes | 1.88 (1.62–2.18)* | 1.55 (1.28–1.89)* | 0.90 (0.66–1.23) | 0.95 (0.64–1.40) |
| - Ref. No |  |  |  |  |
| Participation in community groups |  |  |  |  |
| - Yes | 1.11 (0.96–1.28) | 1.08 (0.92–1.27) | 1.65 (1.22–2.23)* | 1.58 (1.11–2.26)* |
| - Ref. No |  |  |  |  |
| Active in religious or spiritual group |  |  |  |  |
| - Yes | 0.83 (0.67–1.02) | 0.92 (0.73–1.16) | 1.30 (0.83–2.02) | 1.38 (0.81–2.36) |
| - Ref. No |  |  |  |  |

OR = Odds ratio, CI = Confidence interval, Non-F2F = Non-face-to-face, SEIFA = Socio-Economic Index for Areas, WHO = World Health Organisation, **Statistically significant at 5% level of significance

**Table S6** Association of the interaction between significant predictors and remoteness with adolescent help-seeking behaviours in Australia(N = 4,837)

| **Interaction between significant predictors and remoteness** | **OR (95% CI)** | **p-value** |
| --- | --- | --- |
| **Formal help-seeking** |  |  |
| Sex# remoteness, |  |  |
| - Male vs inner regional | 1.00 (0.72–1.38) | 0.994 |
| - Male vs outer regional and remote | 0.67 (0.43–1.05) | 0.081 |
| Primary carer’s financial hardship # remoteness |  |  |
| - Yes vs inner regional | 1.70 (1.05–2.78) | 0.032 |
| - Yes vs outer regional and remote | 1.70 (0.99–2.93) | 0.054 |
| Ongoing anxiety or depression # remoteness |  |  |
| - Yes vs inner regional | 1.24 (0.78–1.96) | 0.358 |
| - Yes vs outer regional and remote | 0.78 (0.39–1.57) | 0.484 |
| Special health care needs # remoteness |  |  |
| - Yes vs inner regional | 1.04 (0.66–1.64) | 0.868 |
| - Yes vs outer regional and remote | 0.93 (0.48–1.81) | 0.836 |
| Suicidal thoughts and behaviours # remoteness |  |  |
| - Yes vs inner regional | 0.89 (0.58–1.36) | 0.582 |
| - Yes vs outer regional and remote | 1.29 (0.78–2.15) | 0.325 |
| Single parenthood # remoteness |  |  |
| - Yes vs inner regional | 1.11 (0.75–1.65) | 0.592 |
| - Yes vs outer regional and remote | 1.14 (0.69–1.90) | 0.613 |
| Relationship with parents # remoteness |  |  |
| - Top 75% - good vs inner regional | 0.98 (0.66–1.45) | 0.907 |
| - Top 75% - good vs outer regional and remote | 0.84 (0.48–1.50) | 0.563 |
| Bullying victimisation # remoteness |  |  |
| - Yes vs inner regional | 1.07 (0.78–1.49) | 0.667 |
| - Yes vs outer regional and remote | 1.34 (0.84–2.16) | 0.222 |
| Discrimination/unfair treatment # remoteness |  |  |
| - Yes vs inner regional | 1.06 (0.75–1.51) | 0.726 |
| - Yes vs outer regional and remote | 1.09 (0.71–1.68) | 0.682 |
| Participation in community groups # remoteness |  |  |
| - Yes vs inner regional | 1.30 (0.92–1.84) | 0.134 |
| - Yes vs outer regional and remote | 0.84 (0.54–1.33) | 0.464 |
|  |  |  |
| **Non-F2F help-seeking** |  |  |
| Age # remoteness |  |  |
| - 18–19 yrs vs inner regional | 0.90 (0.59–1.38) | 0.621 |
| - 18–19 yrs vs outer regional and remote | 1.11 (0.62–1.98) | 0.728 |
| SEIFA # remoteness |  |  |
| - Second quantile vs inner regional | 0.76 (0.37–1.59) | 0.470 |
| - Second quantile vs outer regional and remote | 0.78 (0.33–1.85) | 0.575 |
| - Third quantile vs inner regional | 0.63 (0.33–1.18) | 0.148 |
| - Third quantile vs outer regional and remote | 1.24 (0.54–2.83) | 0.616 |
| - Fourth quantile vs inner regional | 0.68 (0.29–1.56) | 0.361 |
| - Fourth quantile vs outer regional and remote | 1.30 (0.46–3.68) | 0.621 |
| - Fifth quantile vs inner regional | 0.67 (0.20–2.23) | 0.514 |
| - Fifth quantile vs outer regional and remote | 0.55 (0.08–3.56) | 0.526 |
| Ongoing anxiety or depression # remoteness |  |  |
| - Yes vs inner regional | 1.05 (0.58–1.90) | 0.877 |
| - Yes vs outer regional and remote | 0.80 (0.33–1.97) | 0.629 |
| Suicidal thoughts and behaviours # remoteness |  |  |
| - Yes vs inner regional | 0.69 (0.42–1.12) | 0.132 |
| - Yes vs outer regional and remote | 0.75 (0.42–1.35) | 0.336 |
| Social media exposure # remoteness |  |  |
| - 1 to 4 social networks vs inner regional | 0.70 (0.16–3.15) | 0.645 |
| - 1 to 4 social networks vs outer regional and remote | 1.41 (0.12–16.33) | 0.782 |
| - 4+ social networks vs inner regional | 0.90 (0.18–4.61) | 0.900 |
| - 4+ social networks vs outer regional and remote | 0.56 (0.04–7.45) | 0.661 |
| Relationship with parents # remoteness |  |  |
| - Good (top 75%) vs inner regional | 0.90 (0.55–1.46) | 0.662 |
| - Good (top 75%) vs outer regional and remote | 1.59 (0.88–2.88) | 0.127 |
| Discrimination/ unfair treatment # remoteness |  |  |
| - Yes vs inner regional | 0.83 (0.52–1.33) | 0.435 |
| - Yes vs outer regional and remote | 0.75 (0.40–1.41) | 0.373 |
|  |  |  |
| **Informal help-seeking** |  |  |
| Sex# remoteness |  |  |
| - Male vs inner regional | 0.80 (0.34–1.89) | 0.610 |
| - Male vs outer regional and remote | 0.59 (0.18–1.95) | 0.385 |
| Ongoing anxiety or depression # remoteness |  |  |
| - Yes vs inner regional | 3.03 (0.35–26.11) | 0.313 |
| - Yes vs outer regional and remote | 0.66 (0.14–3.07) | 0.600 |
| Ever tried any drugs # remoteness |  |  |
| - Yes vs inner regional | 0.86 (0.27–2.76) | 0.796 |
| - Yes vs outer regional and remote | 0.34 (0.12–1.02) | 0.053 |
| Relationship with parents # remoteness |  |  |
| - Top 75% - good vs inner regional | 1.34 (0.57–3.14) | 0.504 |
| - Top 75% - good vs outer regional and remote | 0.43 (0.17–1.08) | 0.073 |
| Bullying victimisation # remoteness |  |  |
| - Yes vs inner regional | 0.62 (0.24–1.56) | 0.308 |
| - Yes vs outer regional and remote | 0.75 (0.23–2.44) | 0.628 |
| Participation in community groups # remoteness |  |  |
| Yes vs inner regional | 0.90 (0.38–2.11) | 0.804 |
| Yes vs outer regional and remote | 0.37 (0.14–0.97) | 0.044 |
|  |  |  |
| **Any help-seeking sources** |  |  |
| Single parenthood # remoteness |  |  |
| - Yes vs inner regional | 1.09 (0.31–3.82) | 0.894 |
| - Yes vs outer regional and remote | 2.26 (0.61–8.39) | 0.225 |
| Relationship with parents # remoteness |  |  |
| - Top 75% - good vs inner regional | 1.65 (0.45–6.14) | 0.452 |
| - Top 75% - good vs outer regional and remote | 0.42 (0.14–1.22) | 0.111 |
| Bullying victimisation # remoteness |  |  |
| - Yes vs inner regional | 0.40 (0.13–1.26) | 0.116 |
| - Yes vs outer regional and remote | 0.73 (0.20–2.66) | 0.630 |
|  |  |  |

Note: OR = Odds ratio (adjusted), CI = Confidence interval, Non-F2F = Non-face-to-face, SEIFA = Socio-Economic Index for Areas, Reference group: major cities (urban areas)

**References**

1. Aubert, S., et al., *Global matrix 3.0 physical activity report card grades for children and youth: results and analysis from 49 countries.* Journal of physical activity and health, 2018. **15**(s2): p. S251-S273.

2. Bull, F.C., et al., *World Health Organization 2020 guidelines on physical activity and sedentary behaviour.* British journal of sports medicine, 2020. **54**(24): p. 1451-1462.

3. Guo, S., S. Goldfeld, and L. Mundy, *Factors that impact mental health help‐seeking in Australian adolescents: a life‐course and socioecological perspective.* Child and Adolescent Mental Health, 2024. **29**(2): p. 170-180.

4. Gray, S. and G. Daraganova, *Adolescent help-seeking.* LSAC Annual Statistical Report, 2017. **8**.

5. Pearson, S. and C. Hyde, *Influences on adolescent help-seeking for mental health problems.* Journal of Psychologists and Counsellors in Schools, 2021. **31**(1): p. 110-121.

6. Amos, A., et al., *Remoteness and socioeconomic status reduce access to specialist mental health care across Australia.* Australasian Psychiatry, 2023. **31**(1): p. 19-26.

7. Australian Bureau of Statistics, A.S.G.S. *Remoteness Areas*. 2023; Available from: https://www.abs.gov.au/statistics/standards/australian-statistical-geography-standard-asgs-edition-3/jul2021-jun2026/remoteness-structure/remoteness-areas.

8. Fitzpatrick, S.J., et al., *Suicide in rural Australia: A retrospective study of mental health problems, health-seeking and service utilisation.* PLoS One, 2021. **16**(7): p. e0245271.

9. Kaukiainen, A. and K. Kõlves, *Too tough to ask for help? Stoicism and attitudes to mental health professionals in rural Australia.* Rural Remote Health, 2020. **20**(2): p. 5399.

10. Maybery, D., et al., *An online intervention for 18–25‐year‐old youth whose parents have a mental illness and/or substance use disorder: A pilot randomized controlled trial.* Early Intervention in Psychiatry, 2022.

11. Nicholas, J., et al., *Help-seeking behaviour and the Internet: An investigation among Australian adolescents.* AeJAMH (Australian e-Journal for the Advancement of Mental Health), 2004. **3**(1).

12. Hernan, A., et al., *Healthy minds for country youth: Help-seeking for depression among rural adolescents.* The Australian Journal of Rural Health, 2010. **18**(3): p. 118-124.

13. Rughani, J., F.P. Deane, and C.J. Wilson, *Rural adolescents' help‐seeking intentions for emotional problems: The influence of perceived benefits and stoicism.* The Australian Journal of Rural Health, 2011. **19**(2): p. 64-69.

14. Liddle, S.K., S.A. Vella, and F.P. Deane, *Attitudes about mental illness and help seeking among adolescent males.* Psychiatry research, 2021. **301**: p. 113965.

15. Reupert, A., R. Hine, and D. Maybery, *Working with rural families: Issues and responses when a family member has a mental illness*, in *Handbook of rural, remote, and very remote mental health.*, T.A. Carey and J. Gullifer, Editors. 2021, Springer Nature Switzerland AG: Cham. p. 623-640.

16. Orlowski, S., et al., *A rural youth consumer perspective of technology to enhance face-to-face mental health services.* Journal of Child and Family Studies, 2016. **25**(10): p. 3066-3075.

17. O’Connor, M., et al., *Do risk factors for adolescent internalising difficulties differ depending on childhood internalising experiences?* Social psychiatry and psychiatric epidemiology, 2021. **56**: p. 183-192.

18. Ryan, S.M., et al., *Parent and family factors associated with service use by young people with mental health problems: a systematic review.* Early Interv Psychiatry, 2015. **9**(6): p. 433-46.

19. Young, A.S. and D. Rabiner, *Racial/ethnic differences in parent-reported barriers to accessing children’s health services.* Psychological services, 2015. **12**(3): p. 267.

20. Ma, S.O., et al., *Understanding parental knowledge, attitudes and self-efficacy in professional help-seeking for child anxiety.* Journal of Affective Disorders, 2023. **337**: p. 112-119.

21. Corscadden, L., E.J. Callander, and S.M. Topp, *Disparities in access to health care in Australia for people with mental health conditions.* Aust Health Rev, 2019. **43**(6): p. 619-627.

22. Carr, V.J., et al., *Patterns of service use among persons with schizophrenia and other psychotic disorders.* Psychiatric Services, 2003. **54**(2): p. 226-235.

23. Taylor, R., et al., *Mental health and socio-economic variations in Australian suicide.* Social Science & Medicine, 2005. **61**(7): p. 1551-1559.

24. Stenton, J.-a., D. Best, and B. Roberts, *Social support, group involvement, and well-being among the family and friends of problem drinkers.* Journal of Groups in Addiction & Recovery, 2014. **9**(3): p. 199-221.

25. Azzopardi, P.S., et al., *Health and wellbeing of Indigenous adolescents in Australia: a systematic synthesis of population data.* Lancet, 2018. **391**(10122): p. 766-782.

26. Barnett, L.M., et al., *Active gaming as a mechanism to promote physical activity and fundamental movement skill in children*. 2013, Frontiers Media SA. p. 74.

27. Thomas, G., et al., *Screen-based behaviors in Australian adolescents: Longitudinal trends from a 4-year follow-up study.* Preventive Medicine, 2020. **141**: p. 106258.

28. Yu, M. and J. Baxter, *Australian children’s screen time and participation in extracurricular activities.* The longitudinal study of Australian children, annual statistical report, 2015: p. 99-125.

29. Hamilton, J.L., et al., *Leveraging digital media to promote youth mental health: flipping the script on social media-related risk.* Current treatment options in psychiatry, 2024. **11**(2): p. 67-75.

30. Wakefield, C.E., et al., *Grandparents of children with cancer: A controlled study of distress, support, and barriers to care.* Psycho-Oncology, 2014. **23**(8): p. 855-861.

31. Spears, B.A., et al., *Cyberbullying, help-seeking and mental health in young Australians: Implications for public health.* International journal of public health, 2015. **60**: p. 219-226.

32. Mackay, G.J., T.A. Carey, and B. Stevens, *The insider's experience of long-term peer victimisation.* Journal of Psychologists and Counsellors in Schools, 2011. **21**(2): p. 154-174.

33. Kaiser, S., H. Kyrrestad, and S. Fossum, *Help-seeking behavior in Norwegian adolescents: The role of bullying and cyberbullying victimization in a cross-sectional study.* Scandinavian journal of child and adolescent psychiatry and psychology, 2020. **8**: p. 81.

34. Howard, M., R. Grant, and M. Schmidt, *Healthism, rural individualism and self-help: youth mental health in Northwest Tasmania.* Health Sociol Rev, 2024. **33**(3): p. 342-357.

35. Sullivan, E. and W. Bartik, *What do rural young people want from their mental health service.* Aust J Rural Health, 2023. **31**(6): p. 1072-1082.

36. Stain, H.J., et al., *Impact of rurality and substance use on young people at ultra high risk for psychosis.* Early Interv Psychiatry, 2018. **12**(6): p. 1173-1180.
